# Supplementary material for: Identification of Differential Compositions of Aqueous Extracts of Cinnamomi Ramulus and Cinnamomi Cortex
Source: Molecules. 2023 Feb 21;28(5):2015. doi: 10.3390/molecules28052015 (PMC10004064; doi:10.3390/molecules28052015)
Supplement: Supplementary file 1 [file molecules-28-02015-s001.zip › molecules-2186925-supplementary.pdf]

# Supplementary materials

## Identification of differential compositions of aqueous extracts of Cinnamomi ramulus and Cinnamomi cortex

**Pei Wang [1,2,†], Jun Chi[1,2,†], Hui Guo [1,2], Shun-Xiang Wang [1,2], Jing Wang [1,2], Er-Ping Xu [1,2], Li-Ping Dai [1,2,*], Zhi-Min Wang [2,3*]**

[1] Henan University of Chinese Medicine, Zhengzhou 450046, China
[2] Engineering Technology Research Center for Comprehensive Development and Utilization of Authentic Medicinal Materials in Henan Province, Henan University of Chinese Medicine, Zhengzhou 450046, China
[3] National Engineering Laboratory for Quality Control Technology of Chinese Herbal Medicines, Institute of Chinese Materia Medica, China Academy of Chinese Medical Sciences, Beijing 100700, China
* Correspondence: zhmw123@163.com (Z.-M.W.); liping_dai@hactcm.edu.cn (L.-P.D.); Tel.: +86-18703651652
† These authors contributed equally to this work.

**List of Contents**

Figure S1. Typical total ion chromatograms (TIC) of CR (A) and CC (B) in both positive and negative ion modes……………………………………………..……..........................S1

Figure S2. Mass spectra and structural formulas of all detected compounds……..……….S2

Figure S3. Identified compound structures....………......................................................S17

Figure S4. Extracted ion chromatograms (EIC) of 8 standards..……...............................S17

Figure S5. The $MS^2$ spectra of *trans*-cinnamic acid.................……….........................S18

Figure S6. The $MS^2$ spectra of *trans*-cinnamaldehyde...……….................……............S19

Figure S7. The $MS^2$ spectra of epicatechin……..................……...……………………S19

Figure S8. The $MS^2$ spectra of coumarin....………................……………………………S20

Figure S9. 2D PCA score scatter plot for CR and CC after eliminating outliers…….........S20

Figure S10. 2D OPLS-DA score scatter plot for CR and CC……....................................S20

Figure S11. Validate model of OPLS-DA........................................................................S21

Figure S12. Variable importance of projection (VIP) plot of the OPLS-DA....................S21

Figure S13. HPLC chromatograms for quantitative analyses…........................................S21

Figure S14. Dendrograms of hierarchical cluster analysis (HCA) for CR and CC...........S22

Figure S15. The workflow of compound screening with Compound Discoverer 3.2........S22

Table S1. Optimization of the LC-MS conditions………………………...........…..........S23

Table S2. Compound information with VIP>1……………………...…...………….........S24

Table S3. Results of recovery rate……..………………………...…………..................S25

Table S4. Molecular docking fraction……..…………………………...……….…........S26

Table S5. Information on CR and CC.……………………………...……………..........S27

**Identification of other constituents of CR and CC**

S1. Identification of other phenylpropanoids and phenolic acids…………..….................S28

S2. Identification of other flavonoids…...…………………...……….........................S30

S3. Identification of other coumarin…...…………………...………….........................S31

S4. Identification of other Lignans…...…………………...………….........................S31

S5. Identification of terpenoids…...…………………...…………….........................S31

S6. Identification of organic acids…...…………………...………….........................S32

S7. Identification of others…...…………………...…………………..........................S33

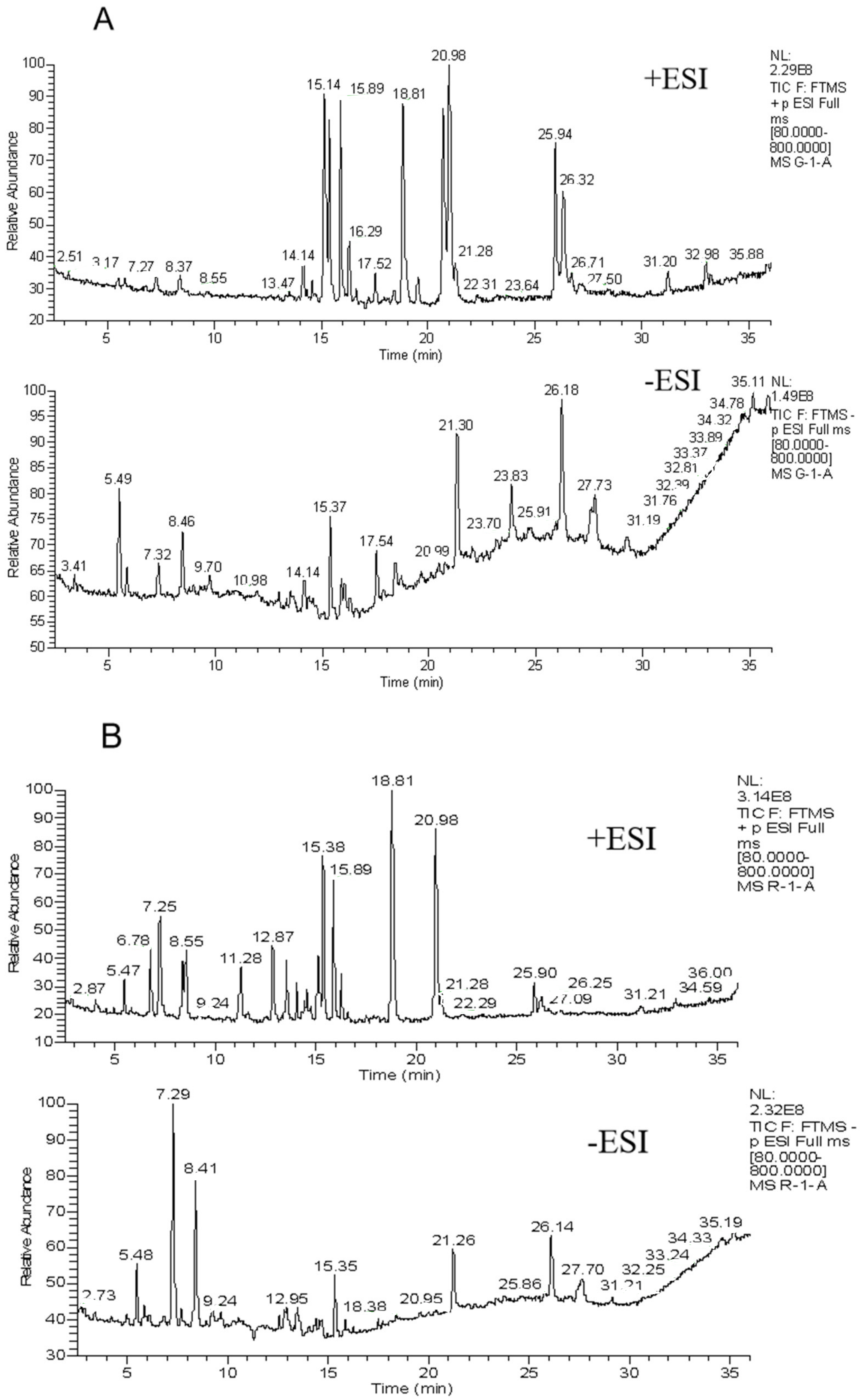


**Figure S1**. Typical total ion chromatograms (TIC) of CR (A) and CC (B) in both positive and negative ion modes.

**Figure S2.** Mass spectra and structural formulas of all detected compounds.

Compound **1**

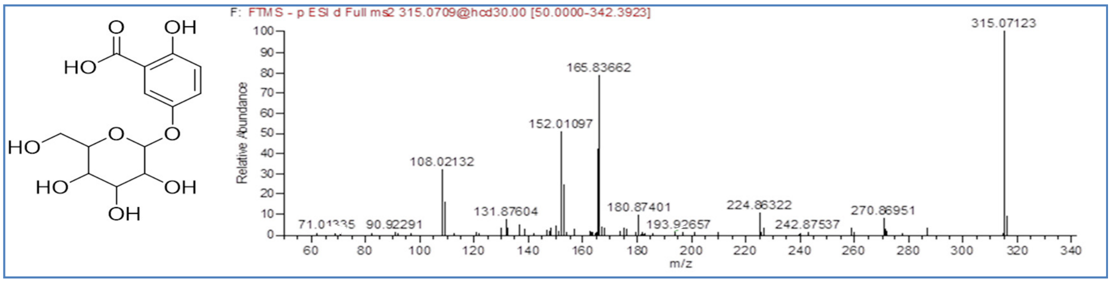


Compound **2**

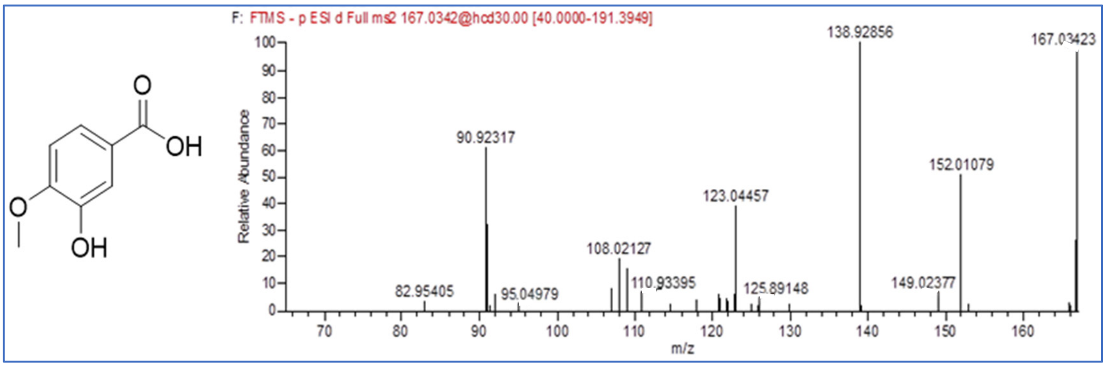


Compound **3**

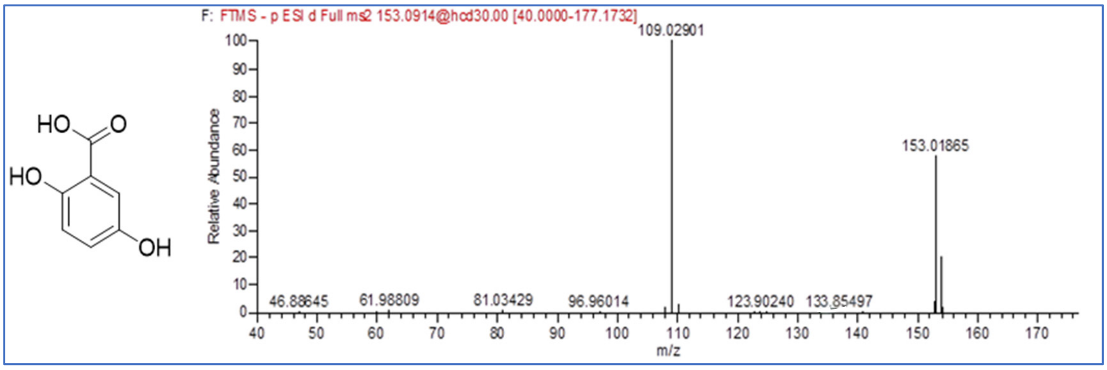


Compound **4**

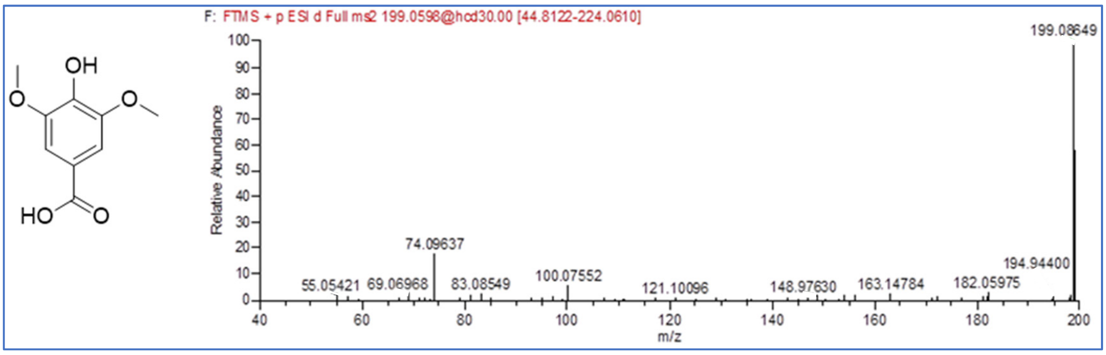

Compound **5**

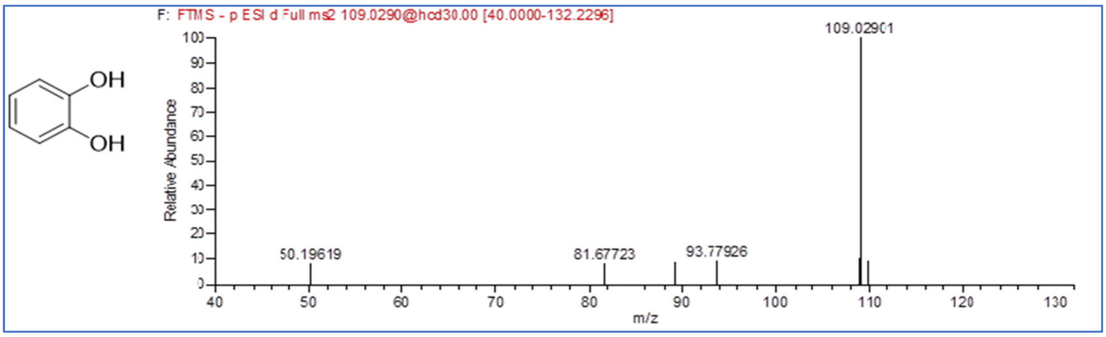


Compound **6**

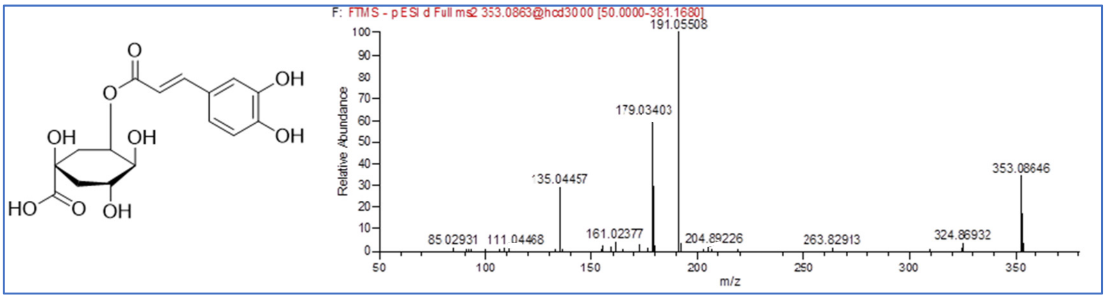


Compound **7**

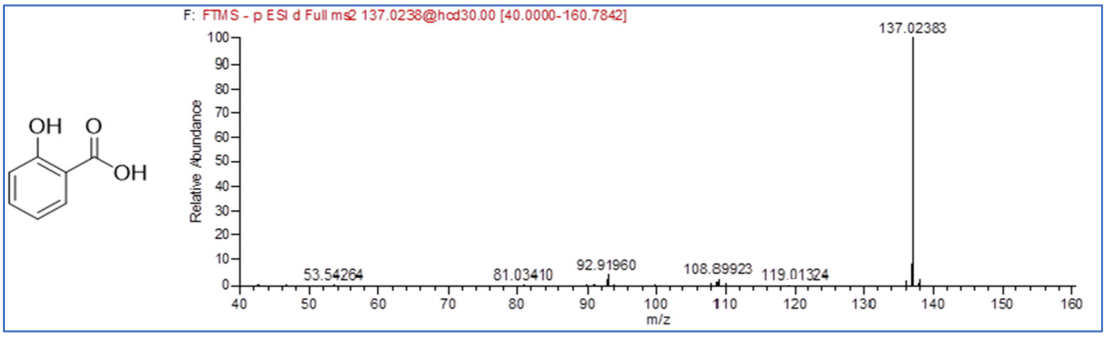


Compound **8**

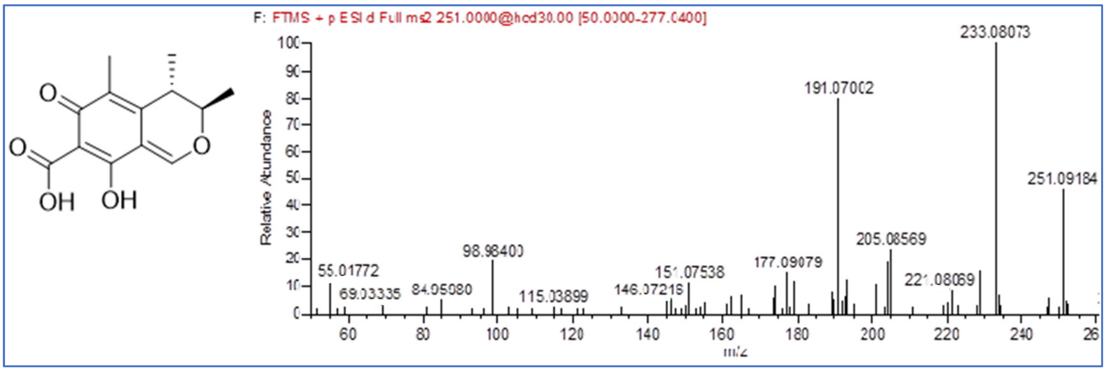

Compound **9**

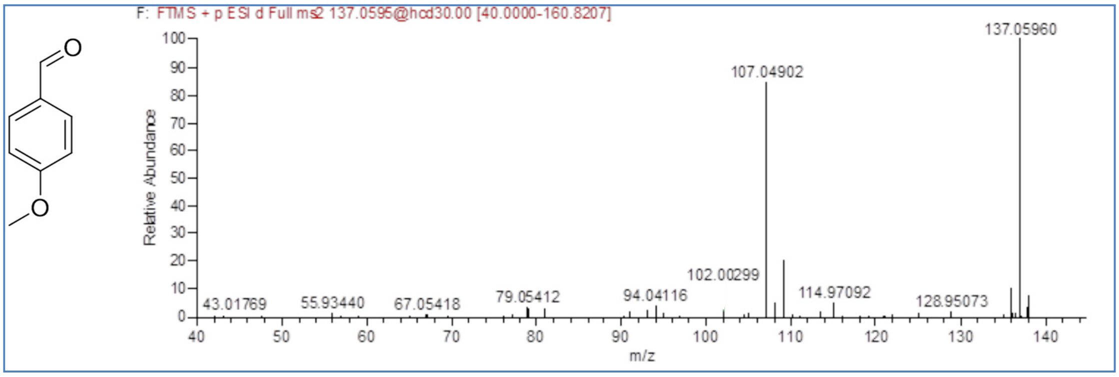


Compound **10**

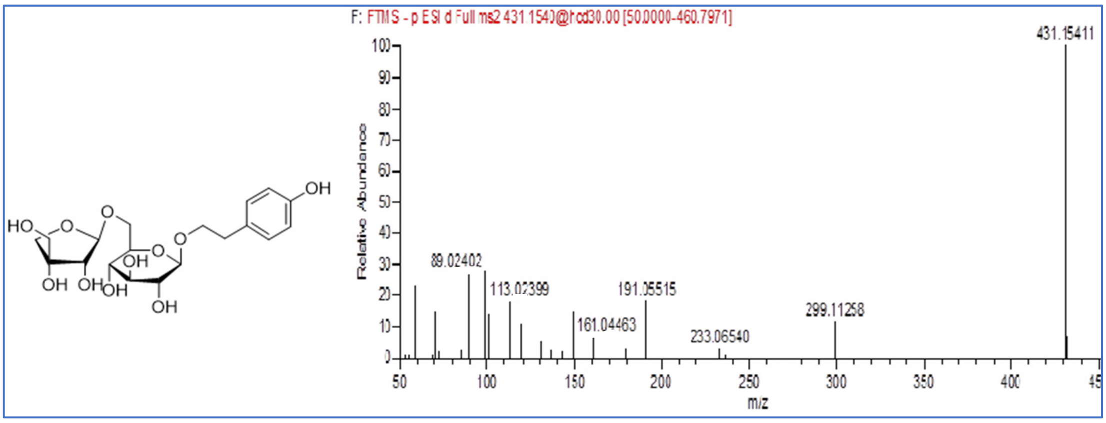


Compound **11**

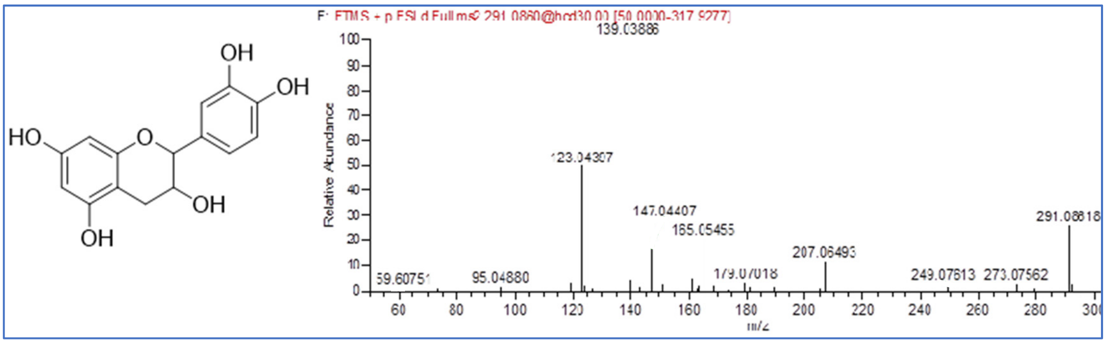


Compound **12**

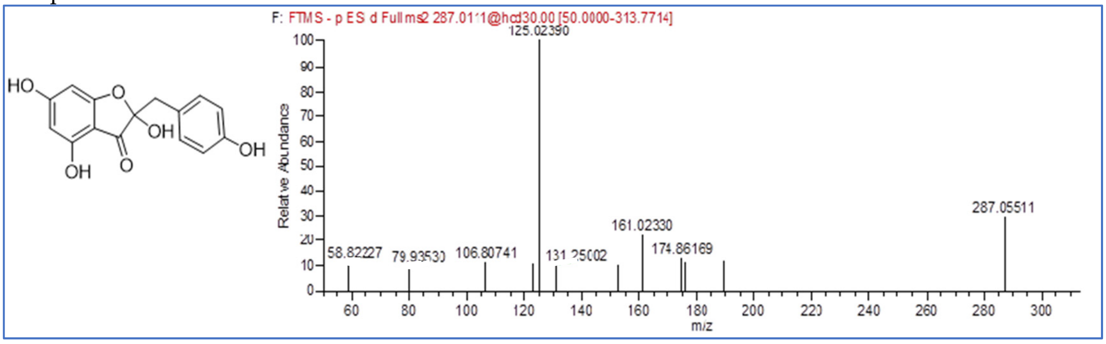

Compound **13**

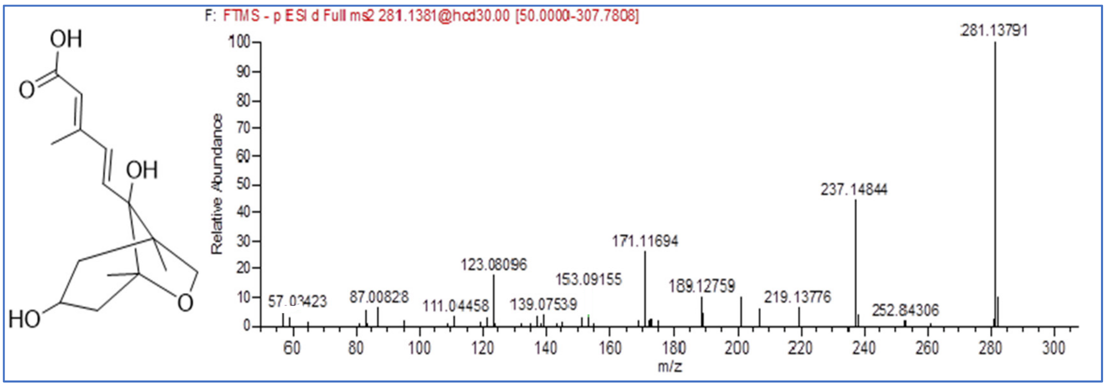


Compound **14**

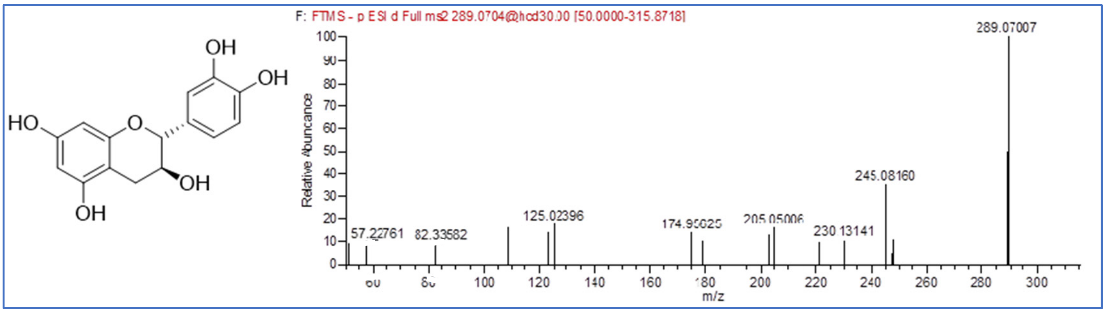


Compound **15**

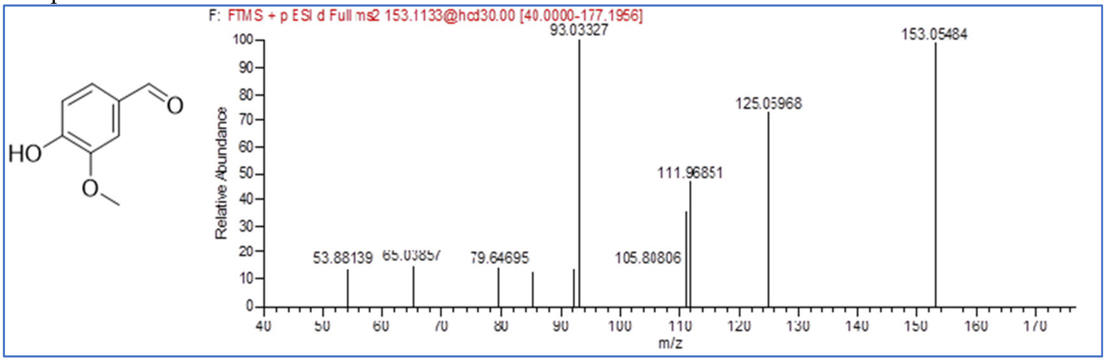


Compound **16**

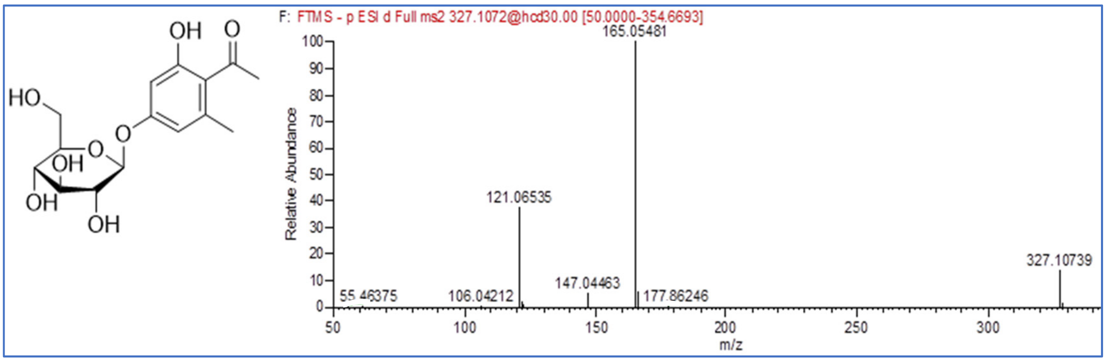

Compound **17**

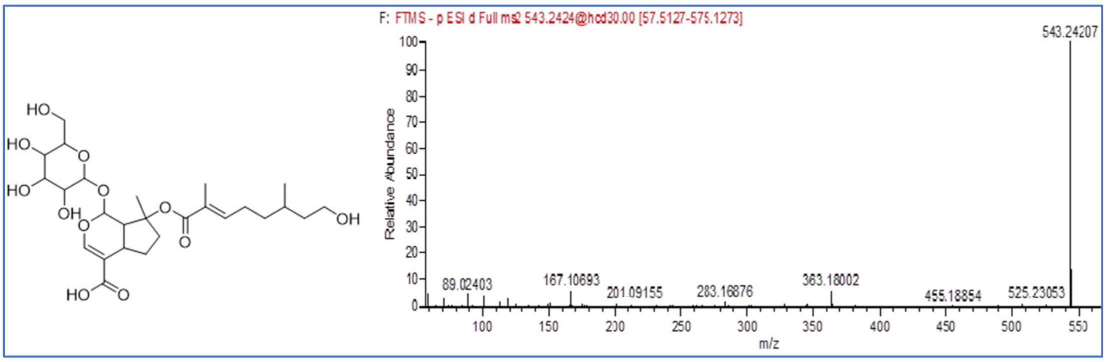


Compound **18**

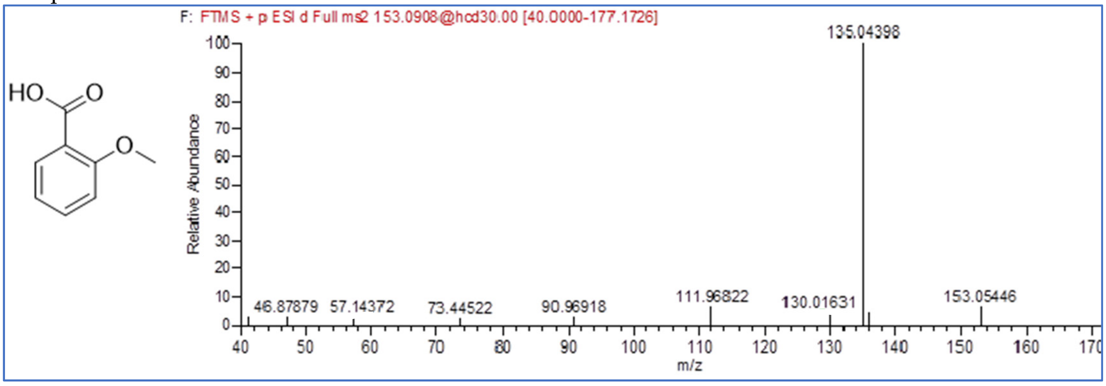


Compound 19

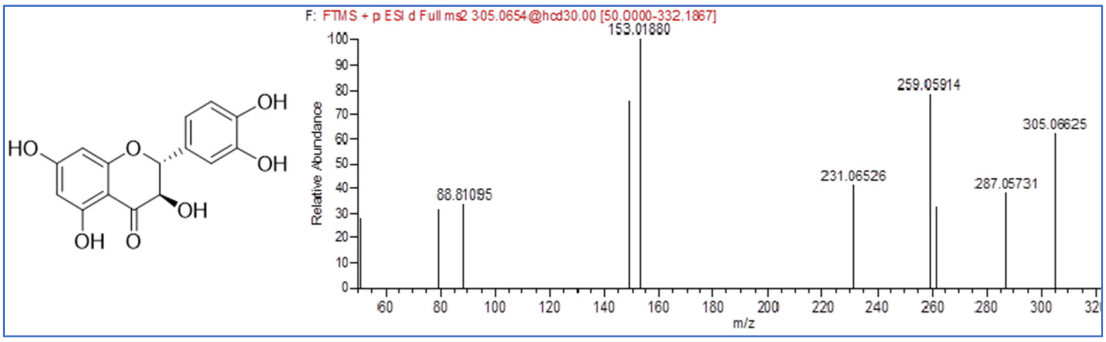


Compound 20

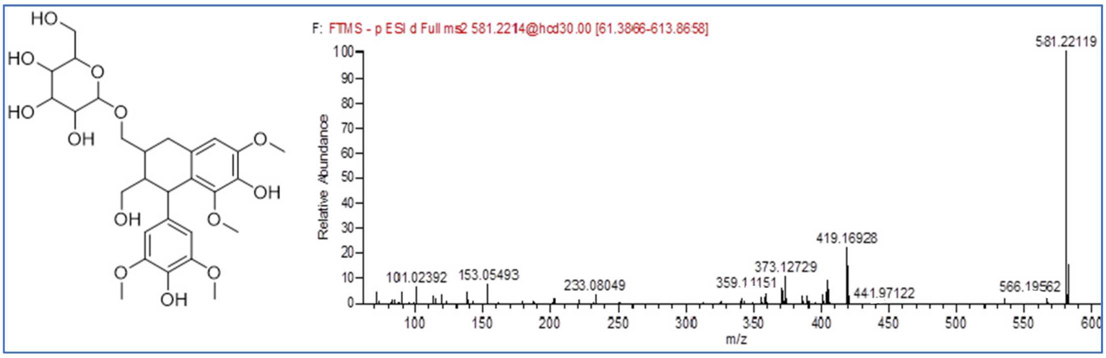

Compound **21**

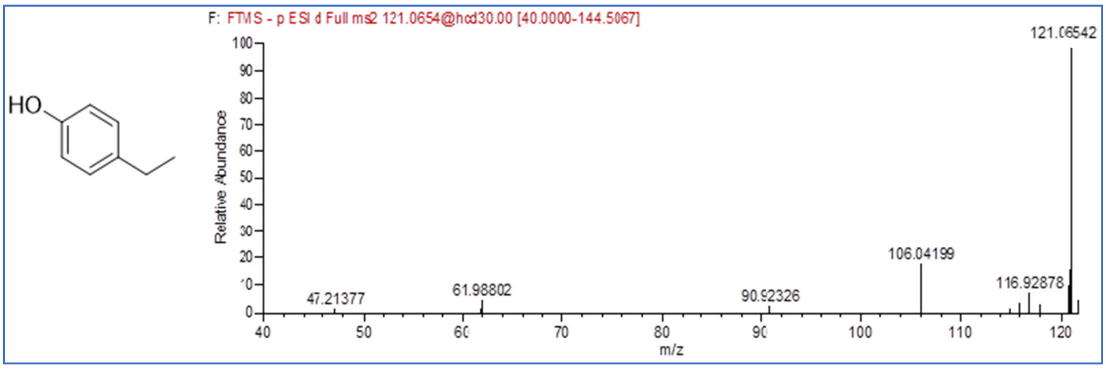


Compound 22

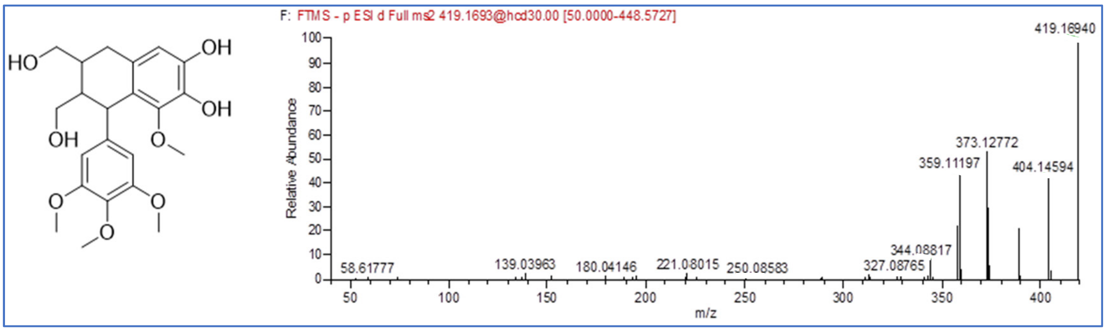


Compound 23

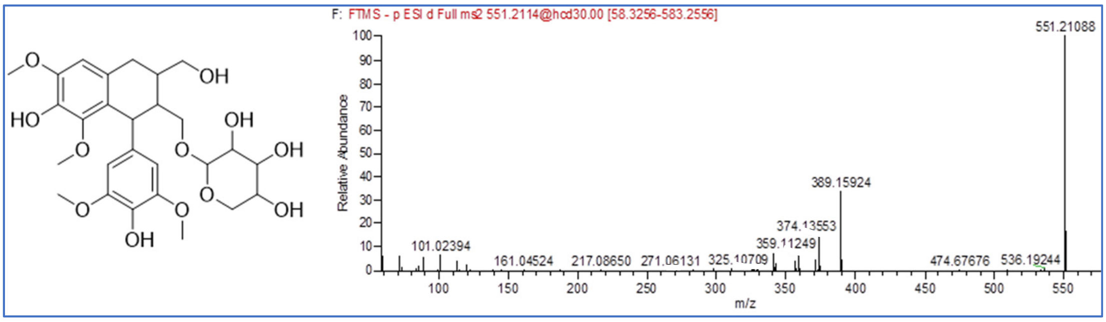


Compound 24

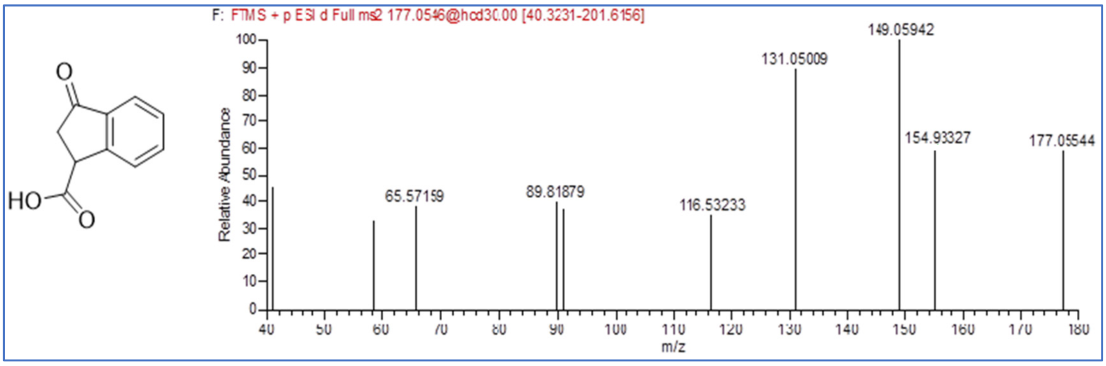

Compound **25**

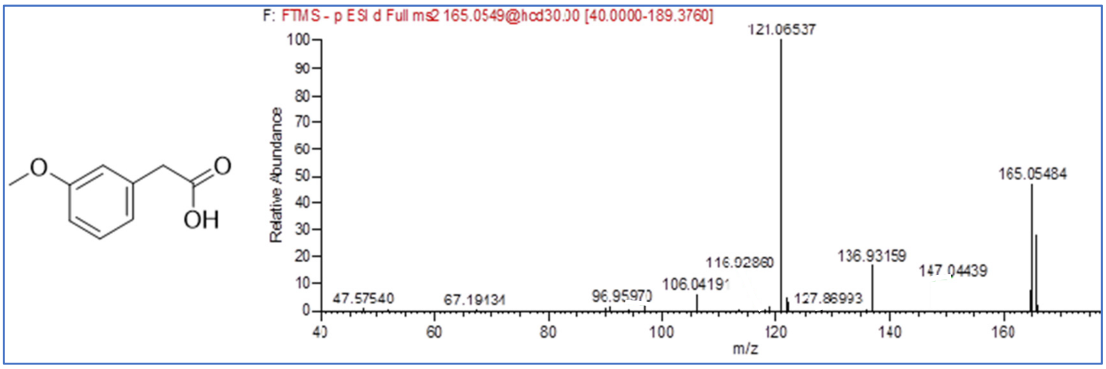


Compound **26**

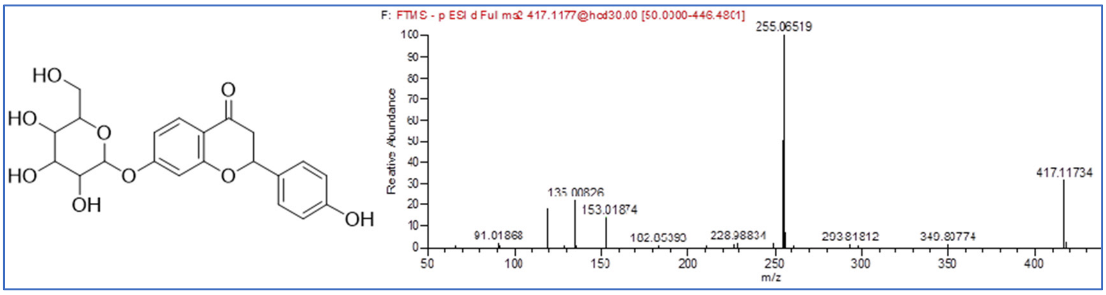


Compound **27**

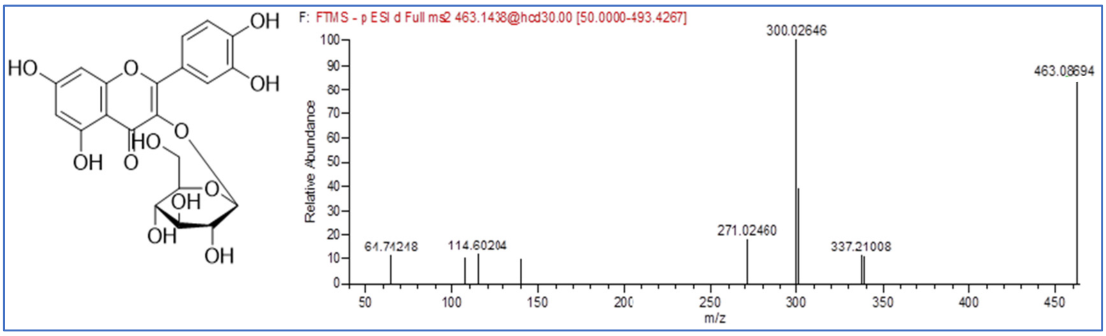


Compound **28**

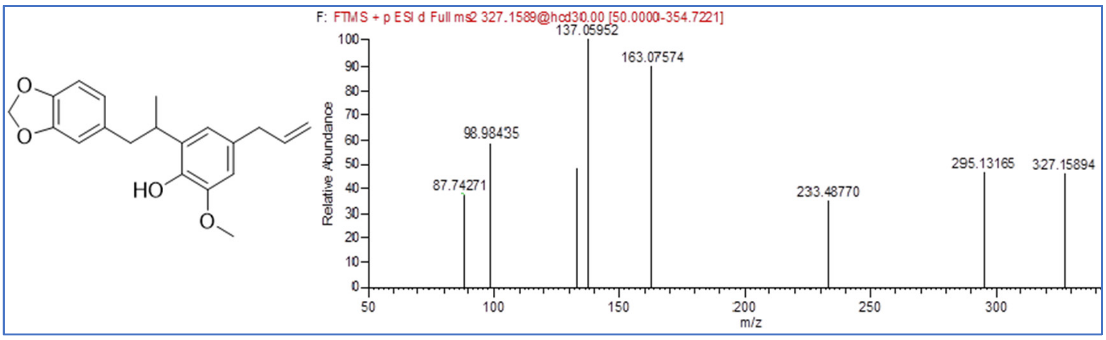

Compound **29**

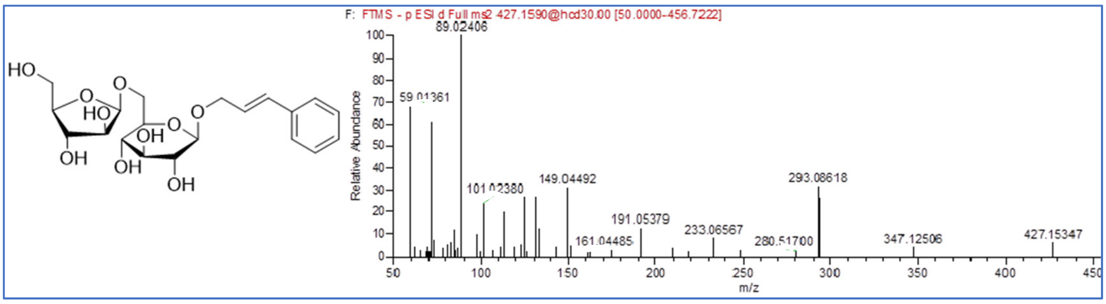


Compound **30**

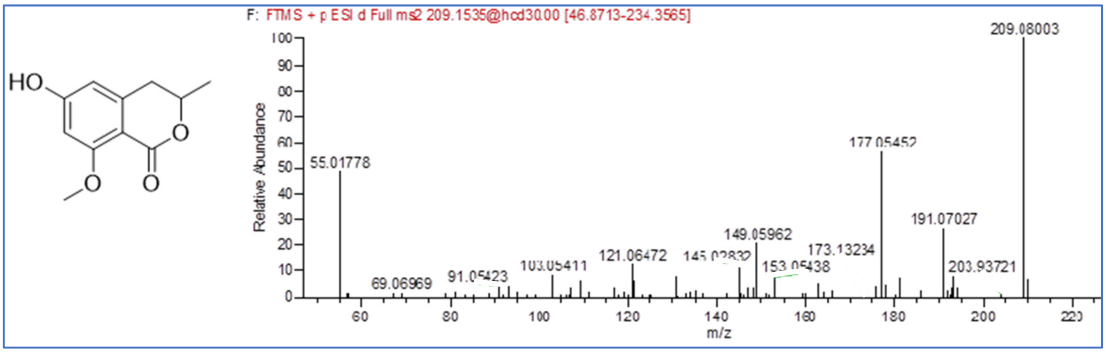


Compound **31**

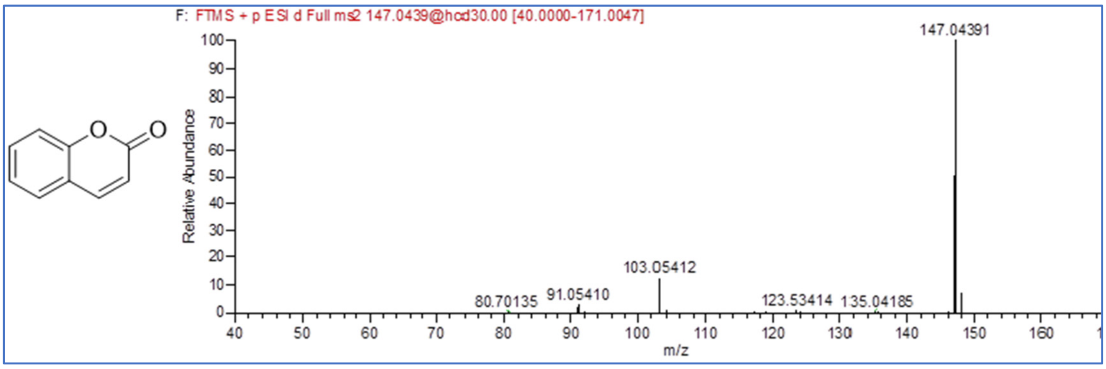


Compound **32**

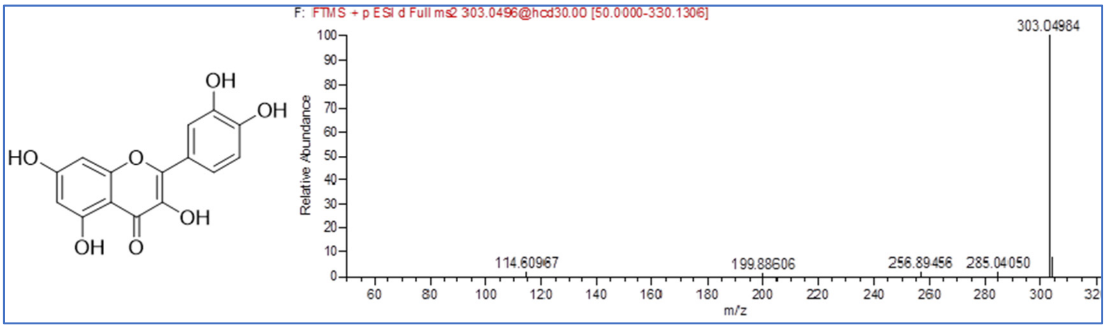

Compound **33**

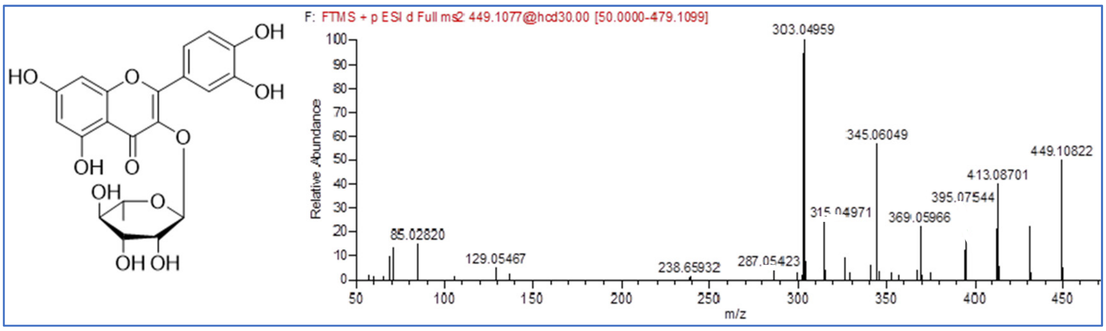


Compound **34**

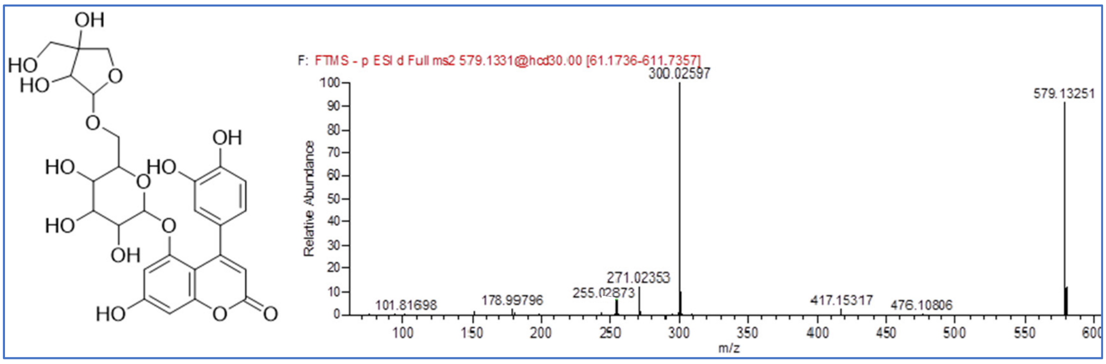


Compound **35**

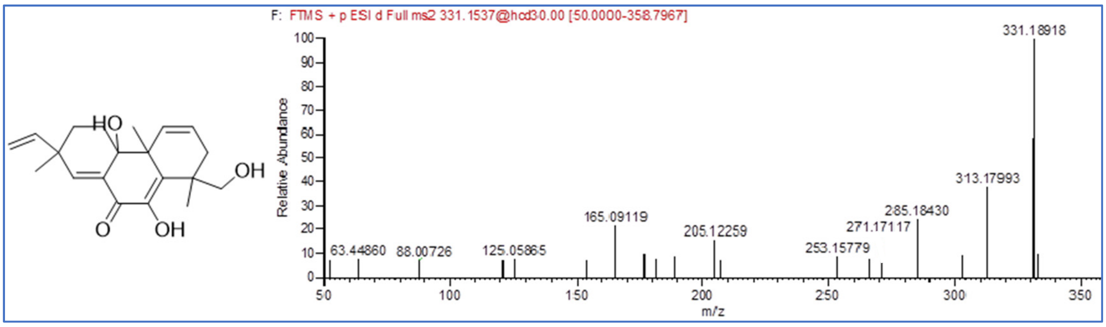


Compound **36**

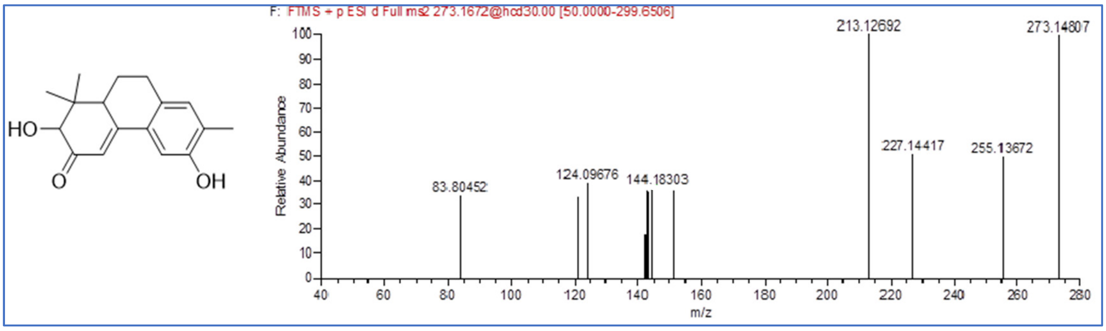

Compound **37**

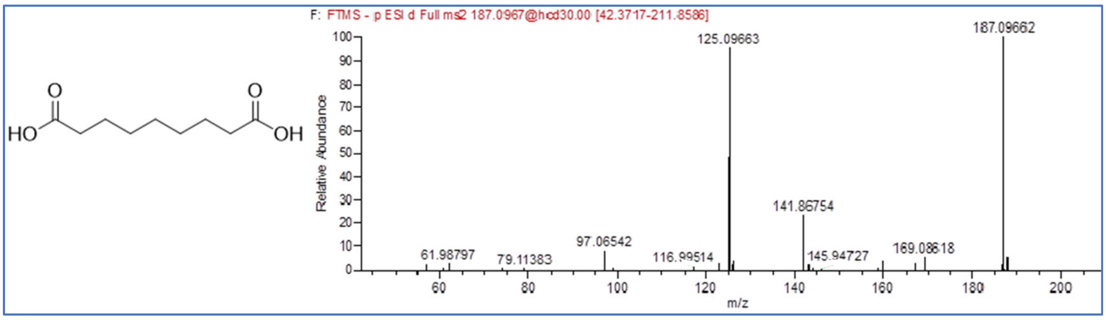


Compound **38**

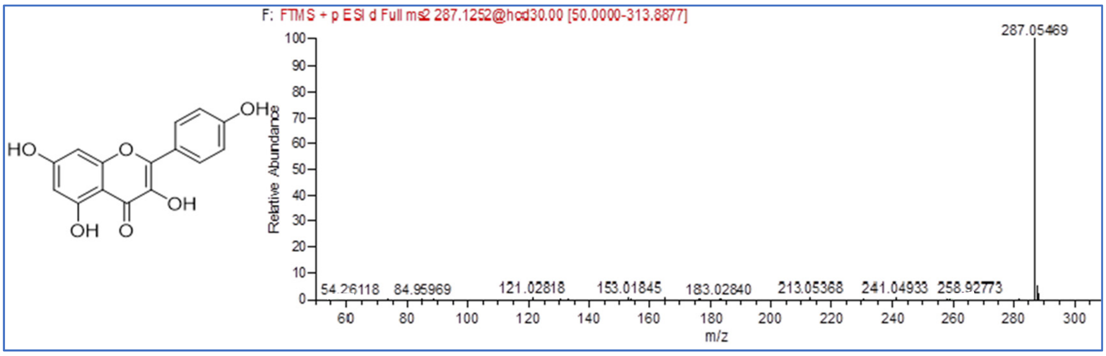


Compound **39**

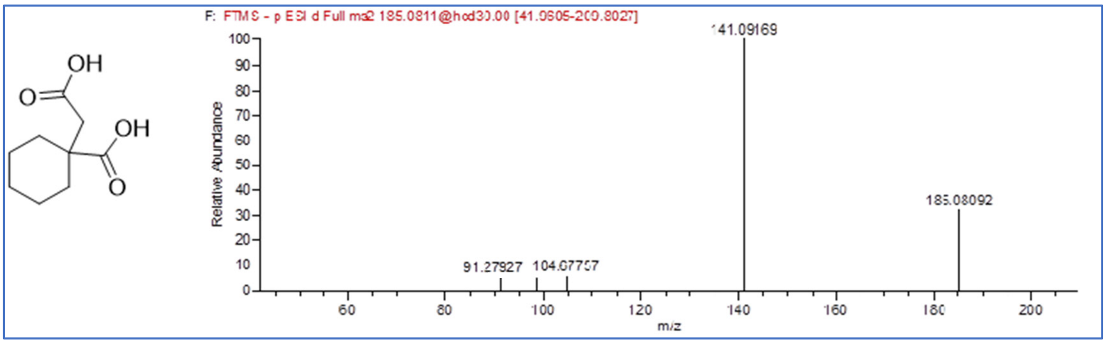


Compound **40**

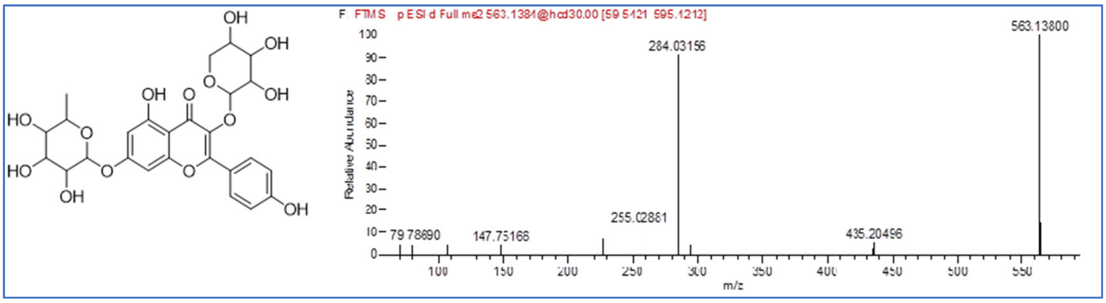

Compound **41**

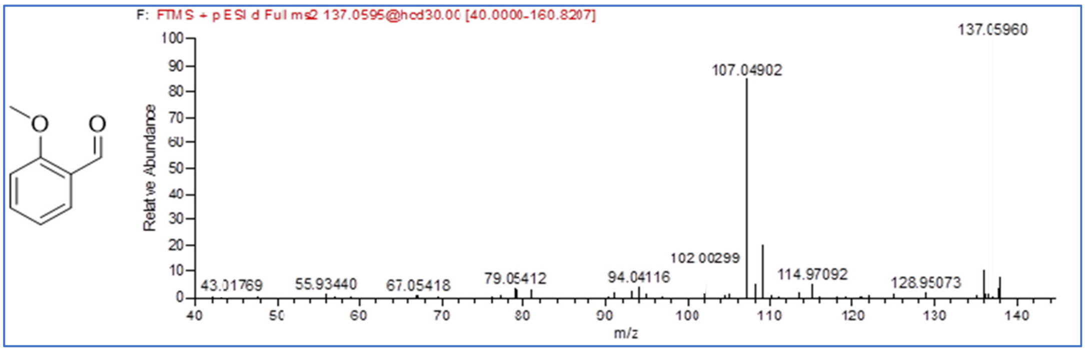


Compound **42**

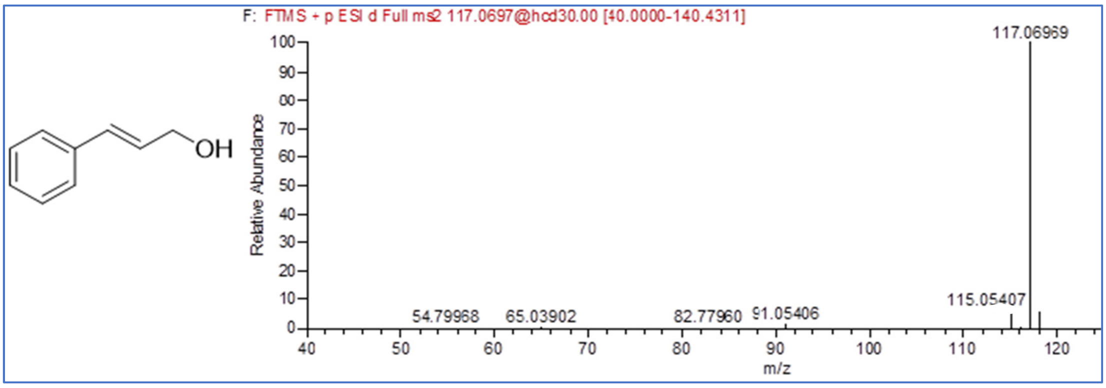


Compound **43**

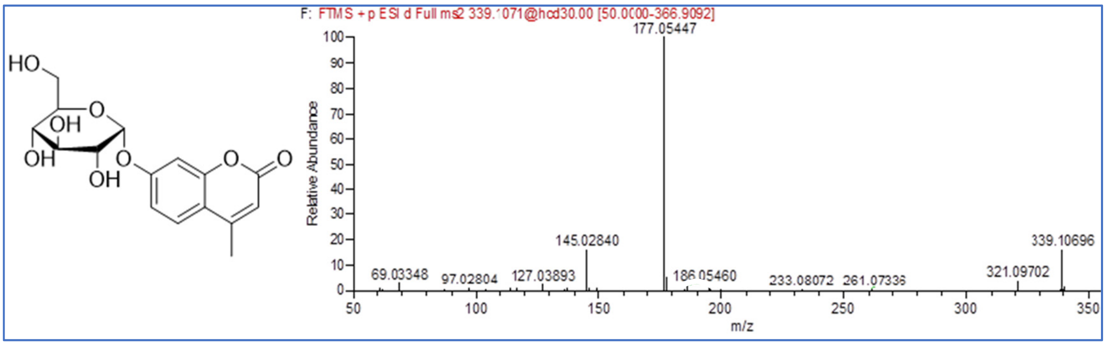


Compound **44**

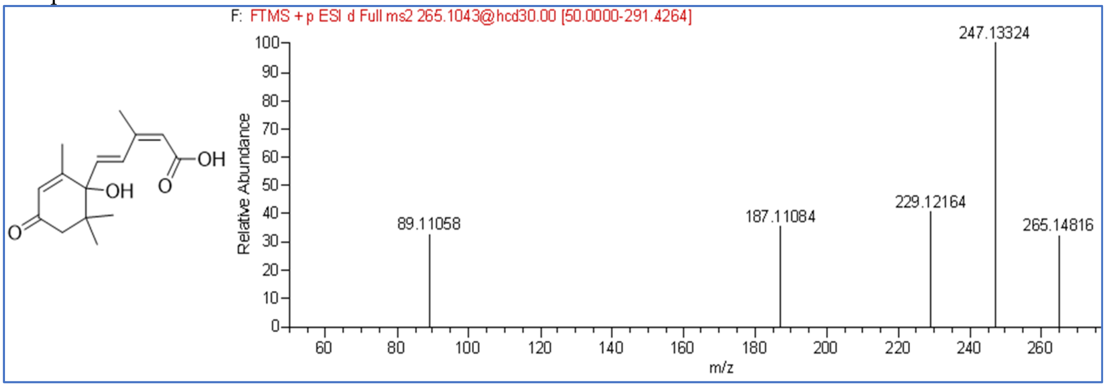

Compound **45**

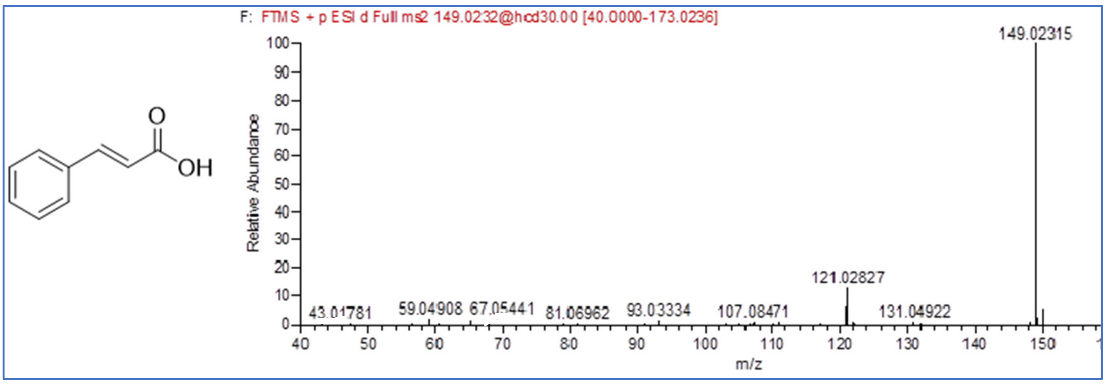


Compound **46**

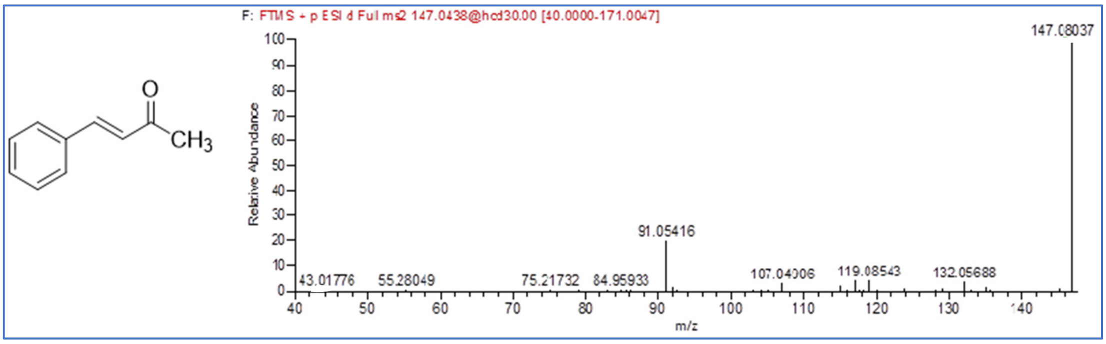


Compound **47**

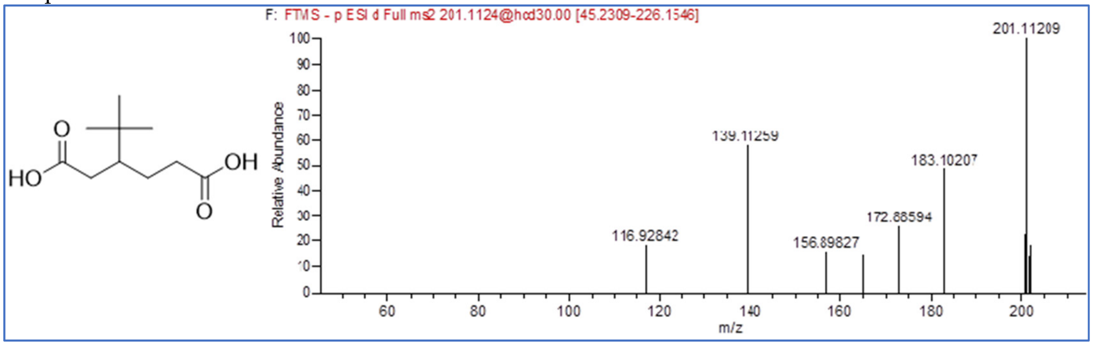


Compound **48**

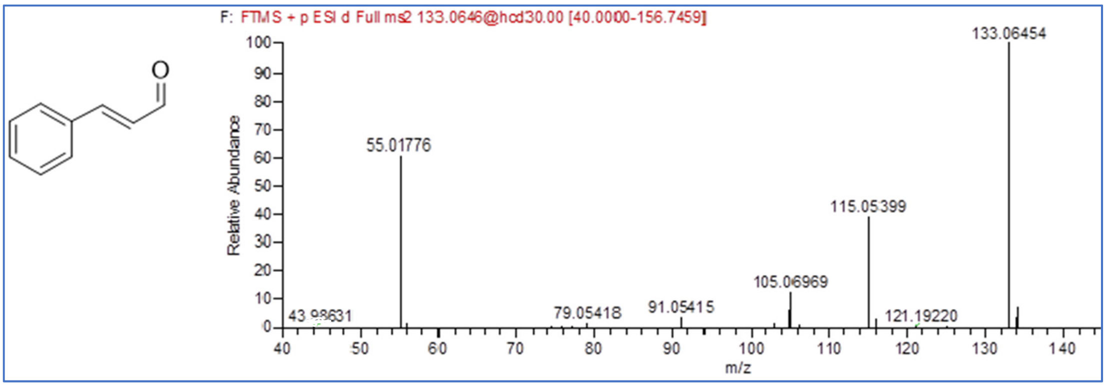

Compound **49**

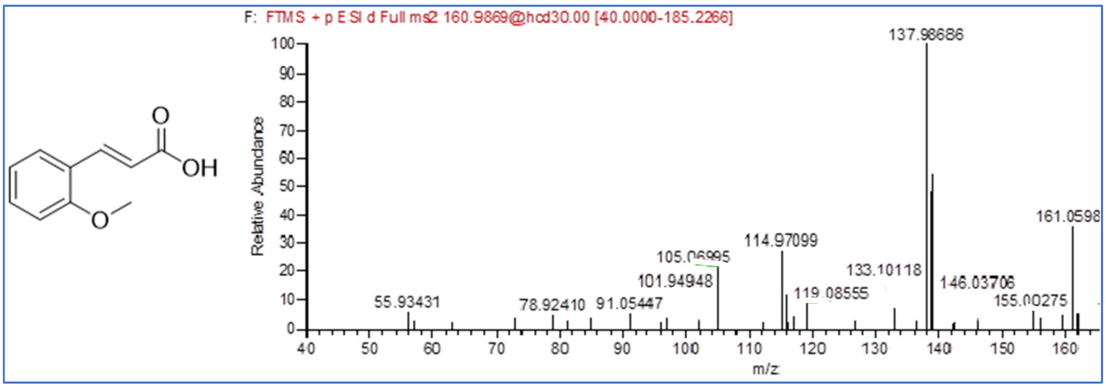


Compound **50**

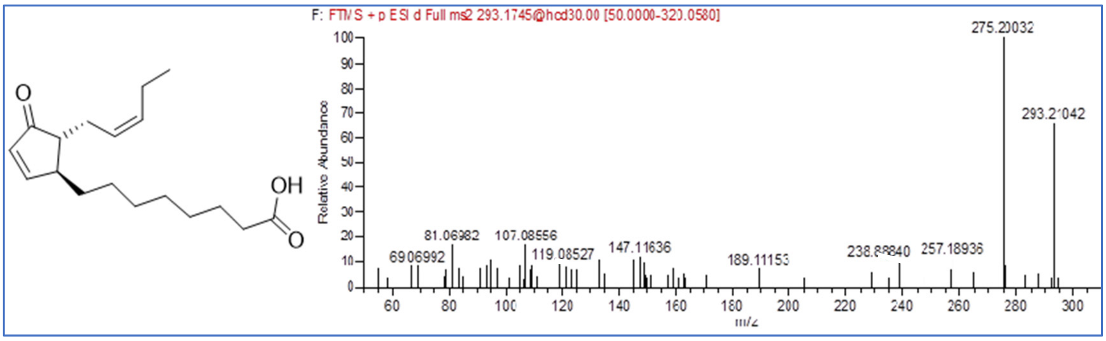


Compound **51**

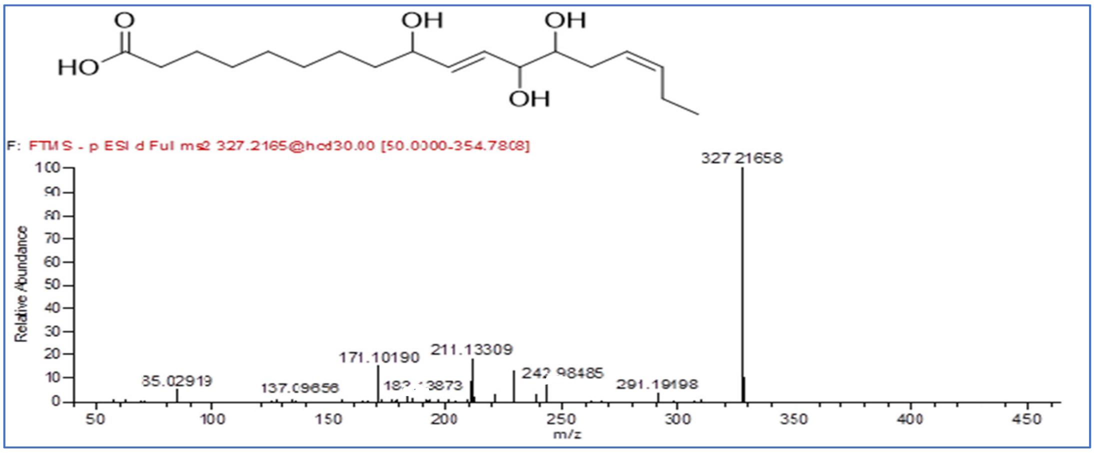


Compound **52**

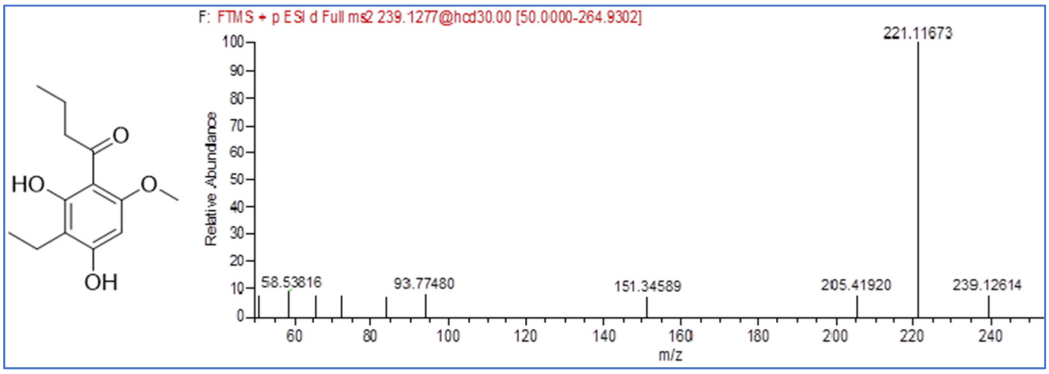

Compound **53**

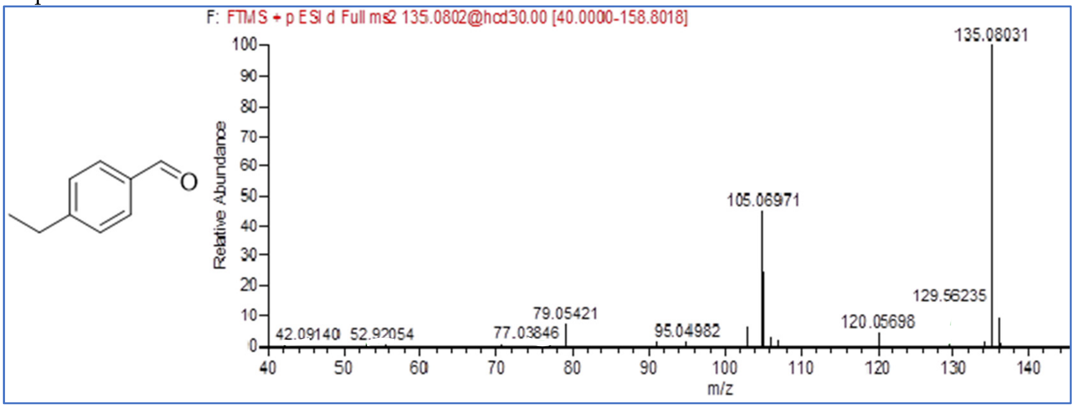


Compound **54**

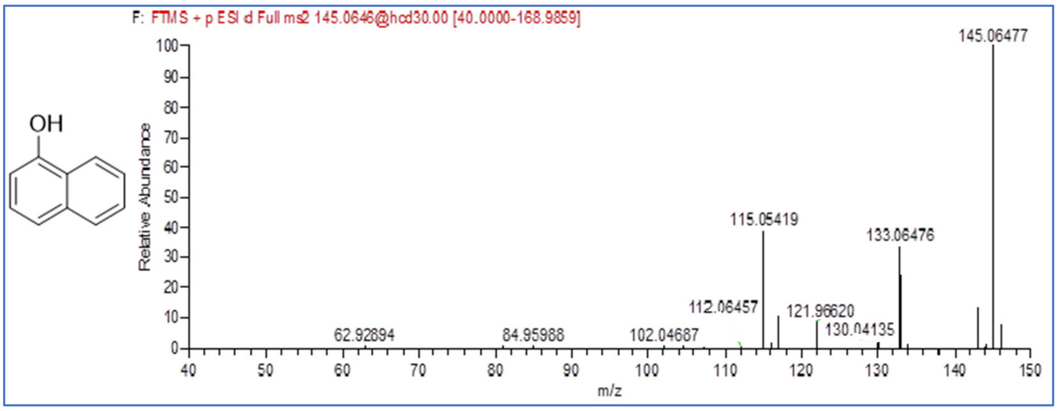


Compound **55**

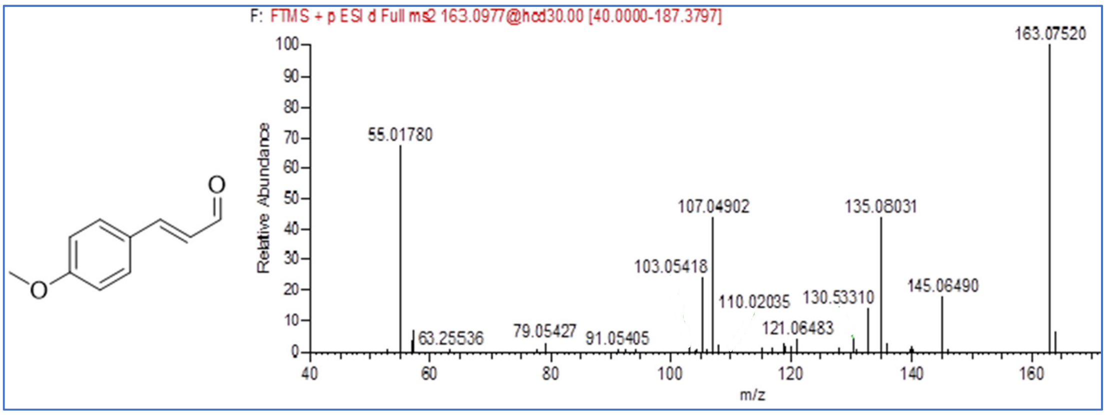

Compound **56**

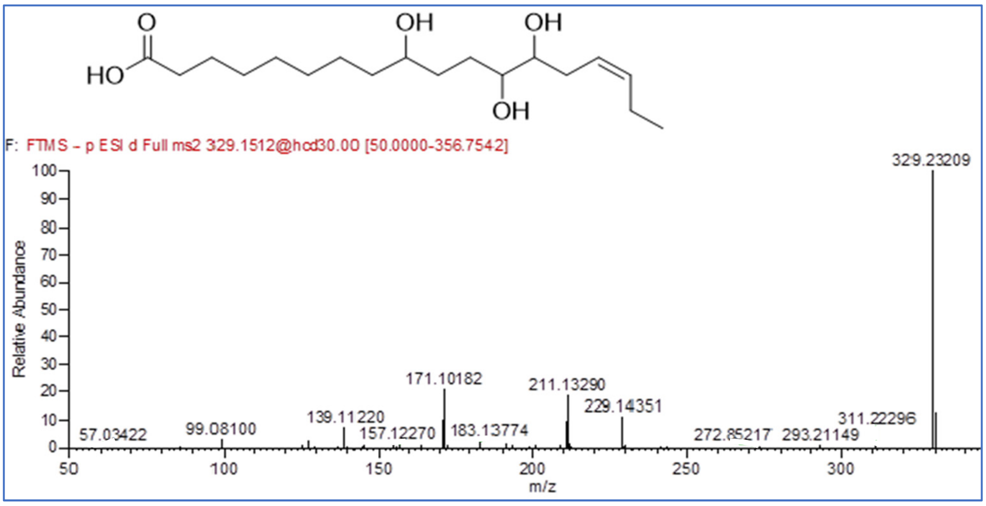


Compound **57**

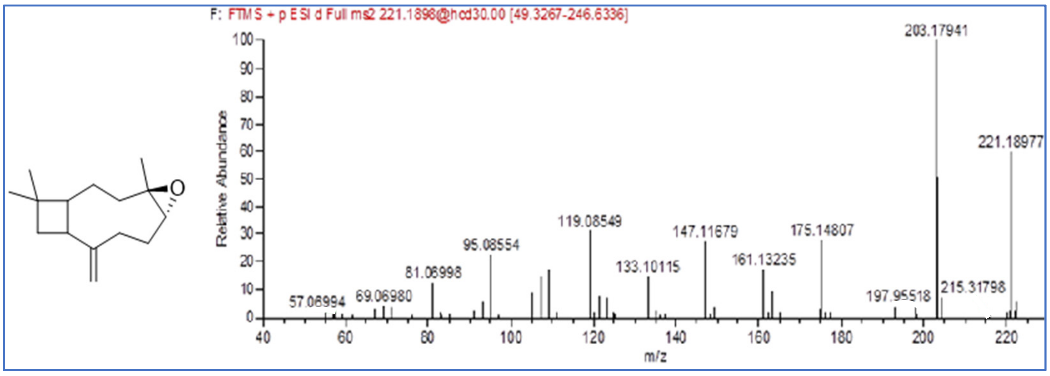


Compound **58**

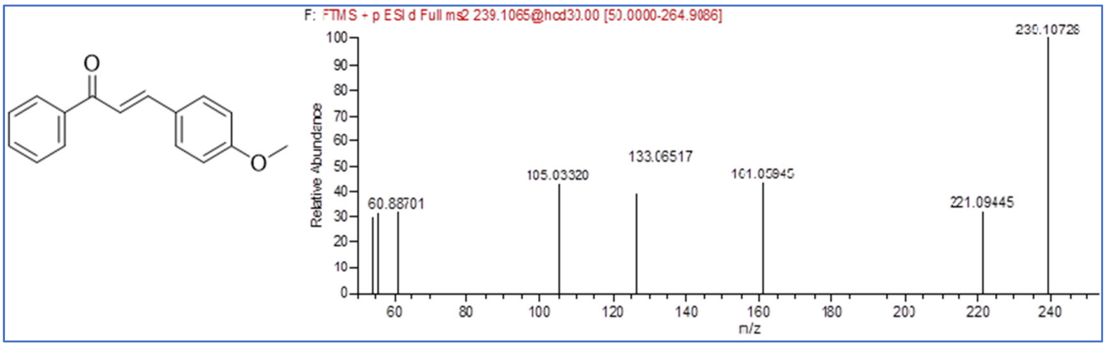

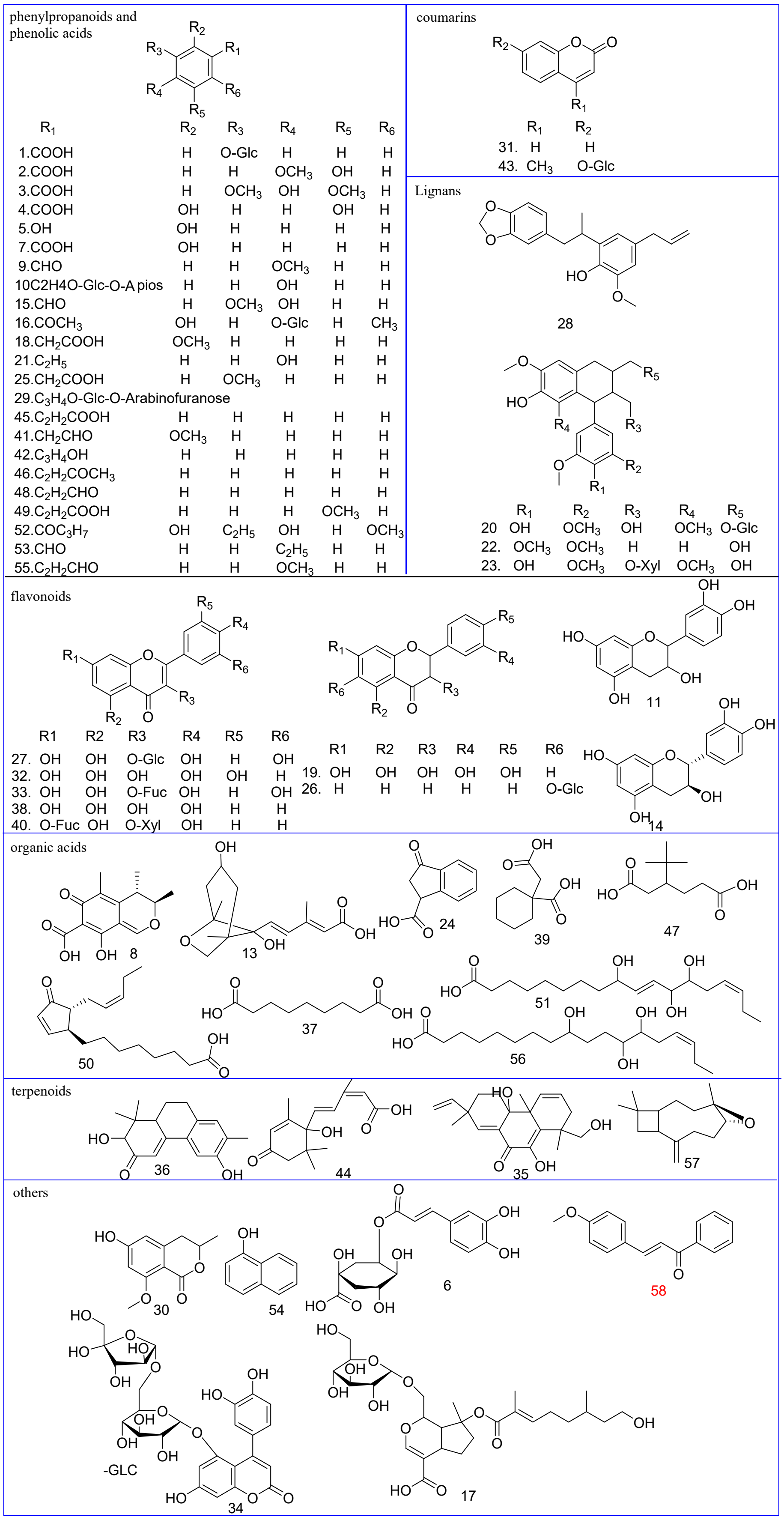


**Figure S3.** Identified compound structures.

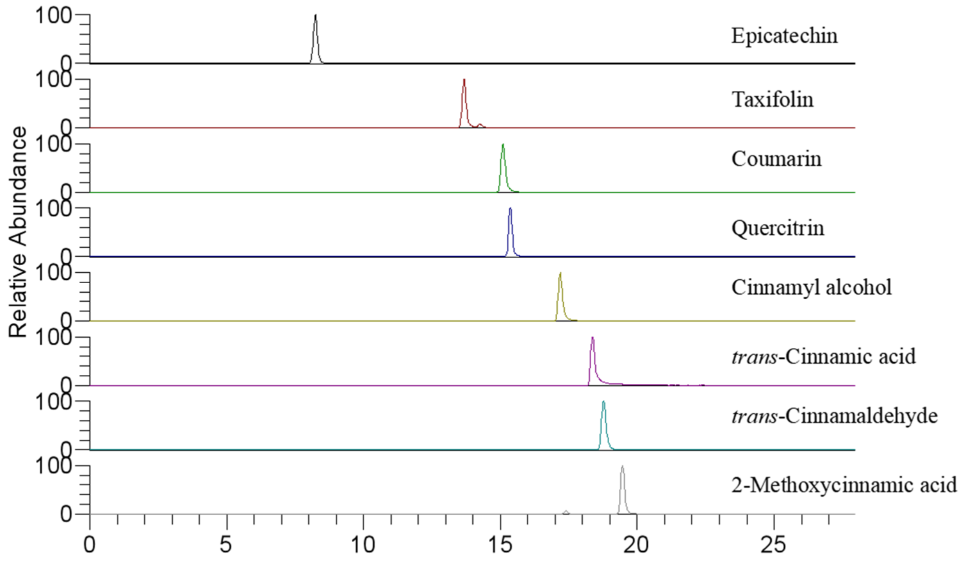


**Figure S4.** Extracted ion chromatograms (EIC) of 8 standards.

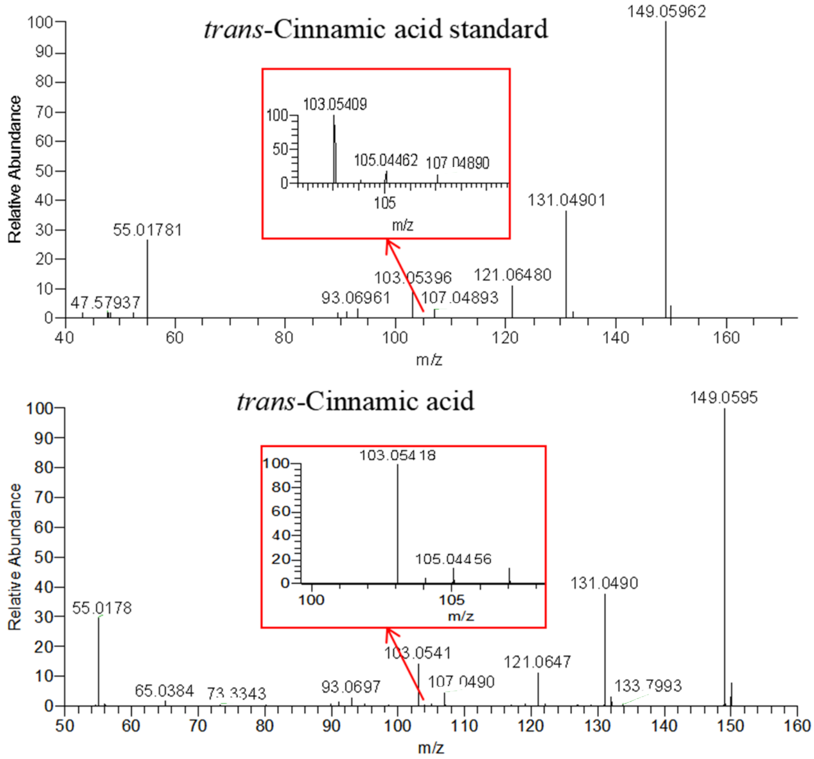


**Figure S5**. The $MS^2$ spectrum of *trans*-cinnamic acid.

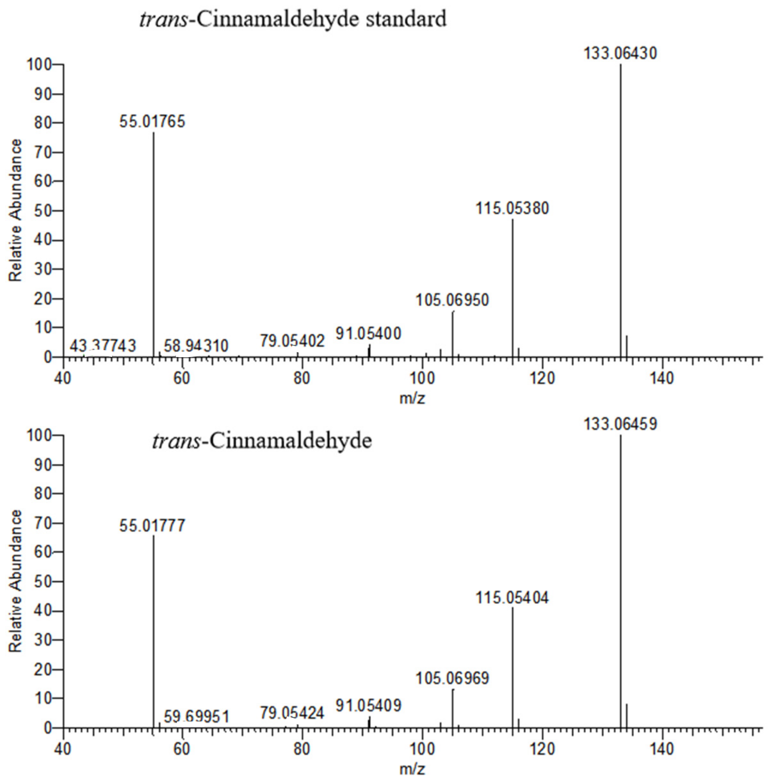


**Figure S6.** The MS² spectrum of *trans*-cinnamaldehyde.

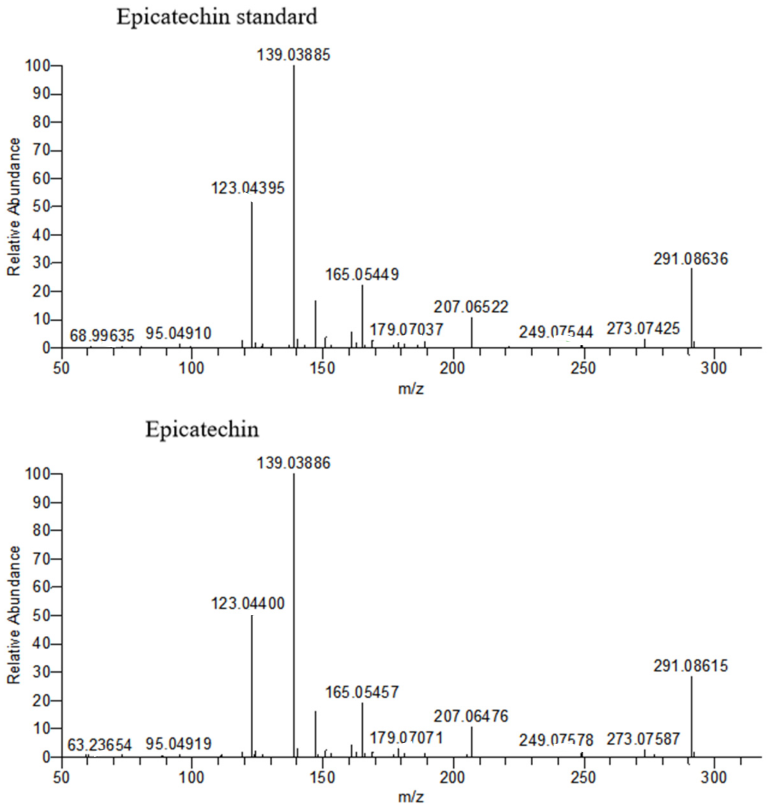


**Figure S7.** The MS² spectrum of epicatechin.

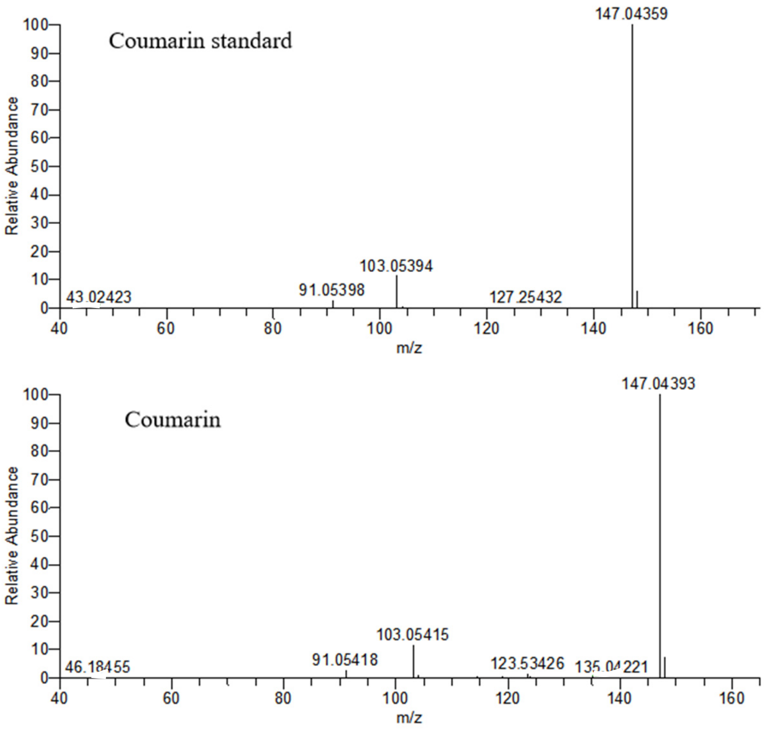


**Figure S8.** The $MS^2$ spectrum of of coumarin.

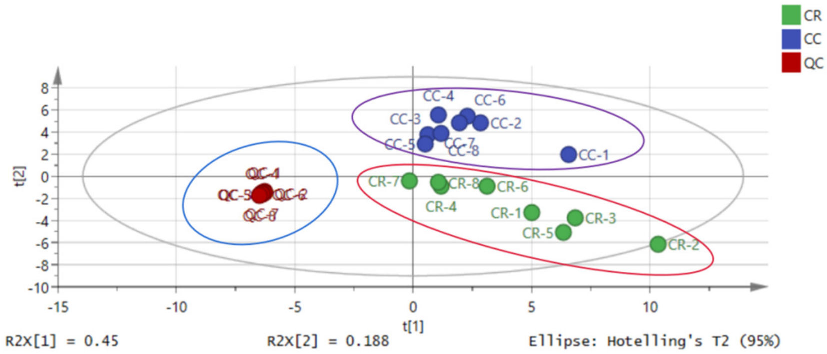


**Figure S9.** 2D PCA score scatter plot for CR, CC and QC after eliminating outliers.

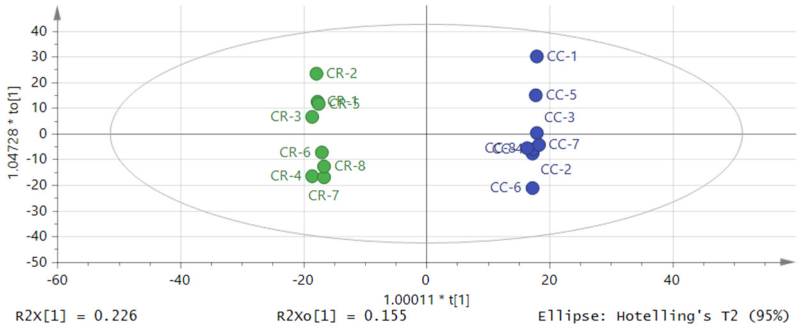


**Figure S10.** 2D OPLS-DA score scatter plot for CR and CC.

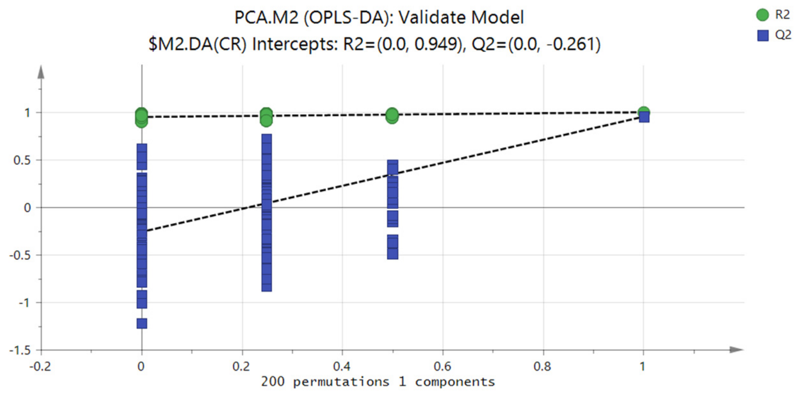


**Figure S11.** Validate model of OPLS-DA.

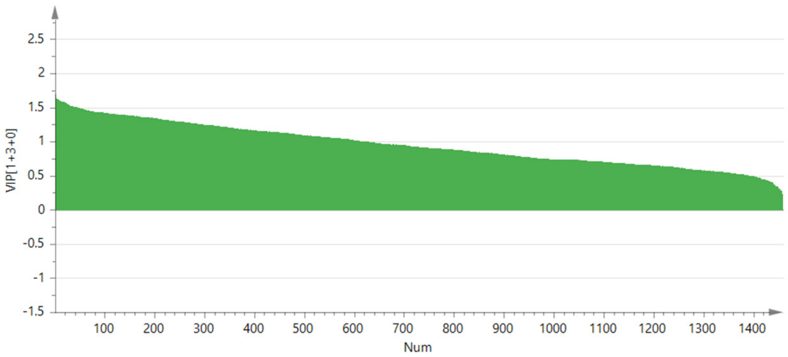


**Figure S12.**Variable importance of projection (VIP) plot of the OPLS-DA.

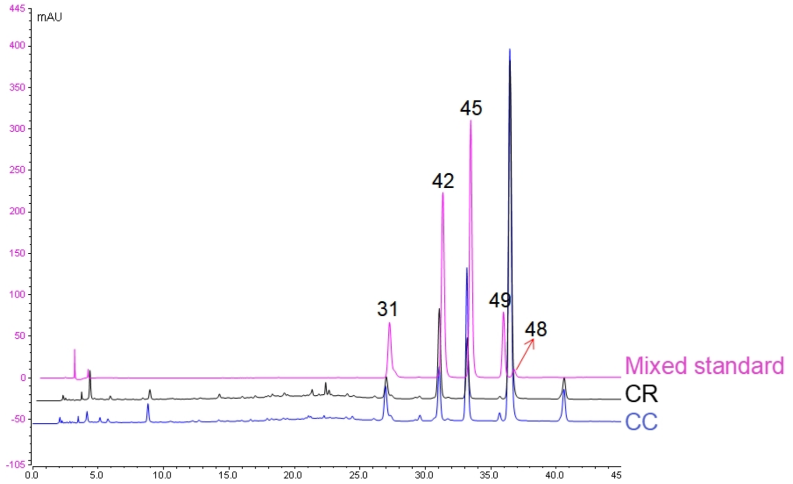


**Figure S13.** HPLC chromatograms for quantitative analyses. **31**: Coumarin, **42**: Cinnamyl alcohol, **45**: *trans*-Cinnamic acid, **49**: 2-Methoxycinnamic acid, **48**: *trans*-Cinnamaldehyde

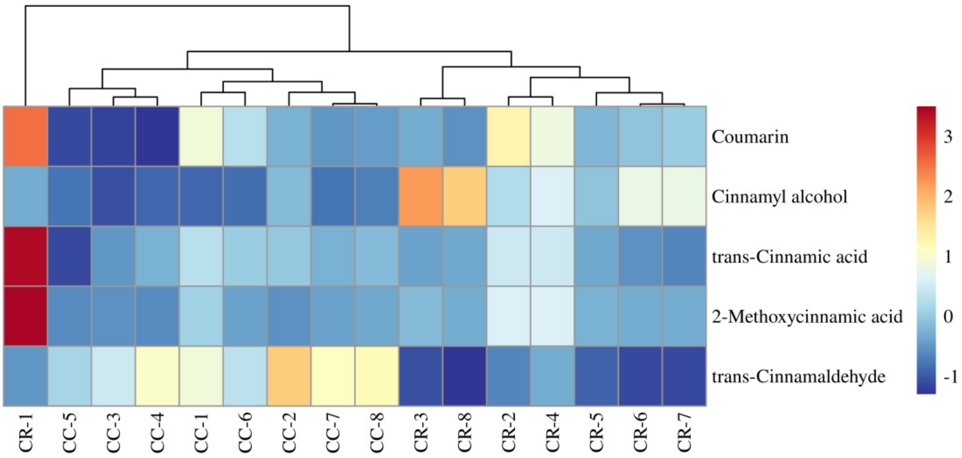


**Figure S14.** Dendrograms of hierarchical cluster analysis (HCA) for CR and CC.

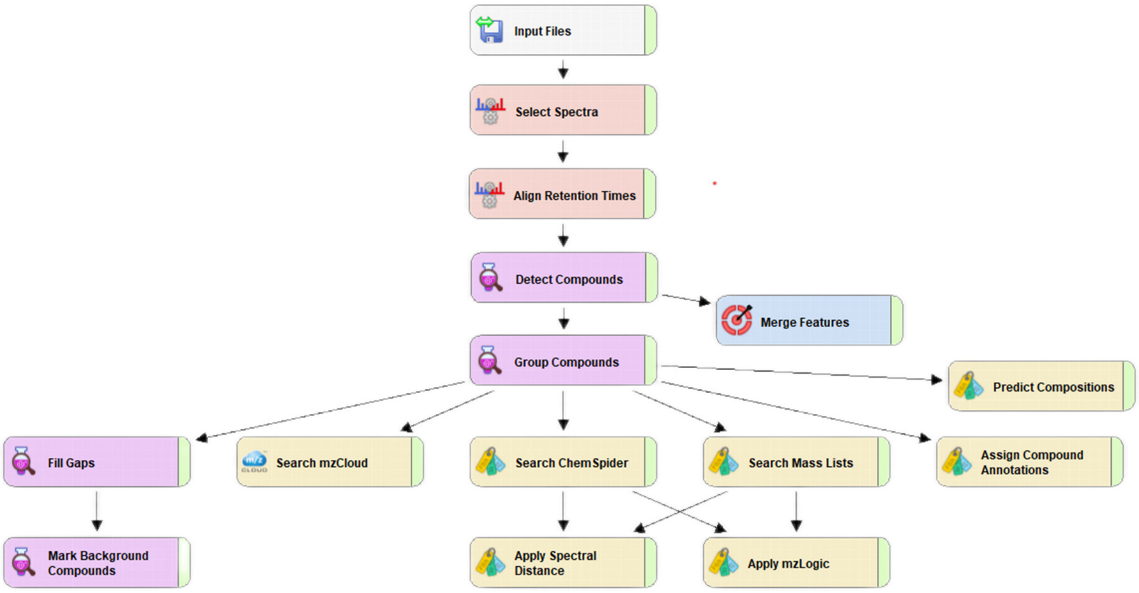


**Figure S15.** The workflow of compound screening with Compound Discoverer 3.2.

**Table S1.** Optimization of the LC-MS conditions

| Condition | The total ion chromatogram of CR | Condition | The total ion chromatogram of CR |
|---|---|---|---|
| Methanol/aqueous formic acid | 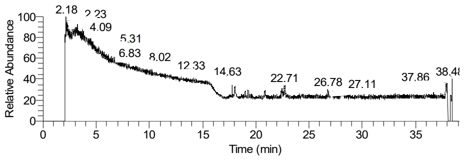 | **Acetonitrile/aqueous formic acid** | 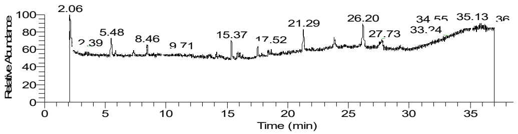 |
| Waters Acquity UPLC BEH C18（2.1 mm×100 mm，1.7 µm） | 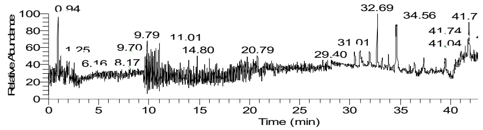 | **HYPERSILGOLD Vanquish C18 (2.1 mm × 100 mm, 1.9 µm)** | 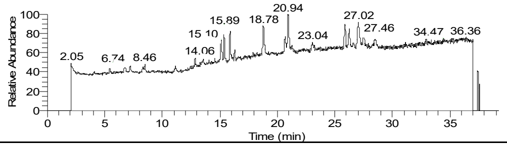 |
| column temperature was set at 35 ℃ | 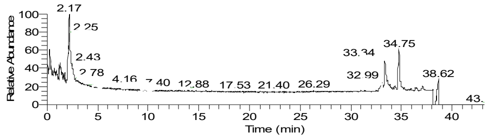 | **column temperature was set at 25 ℃** | 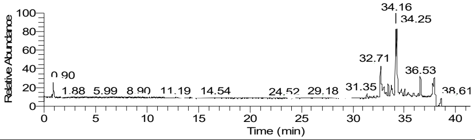 |
| vaporizer temperature was 320 °C | 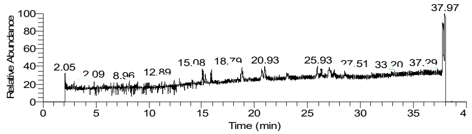 | **vaporizer temperature was 350 °C** | 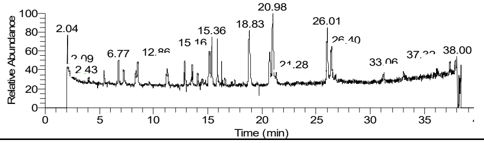 |
| the spray voltage was 3.8 kV(+) | 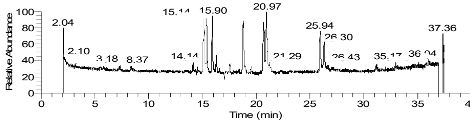 | **the spray voltage was 3.5 kV(+)** | 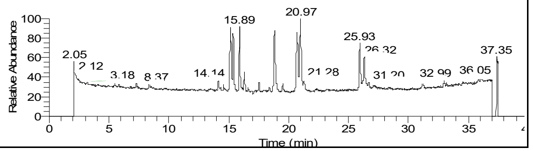 |

**Table S2.** Compound information with VIP>1.

| No. | Name | VIP | Fold change [a] | P-value [b] |
|---|---|---|---|---|
| **3** | Gentisic acid | 1.60199 | -0.93 | 0.0100 |
| **46** | 4-Phenyl-3-buten-2-one | 1.55590 | 3.28 | 0.0001 |
| **55** | 4-Methoxycinnamaldehyde | 1.51806 | / | 0.4002 |
| **24** | 3-Oxoindane-1-carboxylic acid | 1.50622 | 2.93 | 0.0001 |
| **53** | 4-Ethylbenzaldehyde | 1.48790 | Specific in CR | 0.0002 |
| **58** | 4-Methoxychalcone | 1.44618 | 3.26 | 0.0005 |
| **8** | Citrinin | 1.44300 | Specific in CC | 0.0002 |
| **1** | Gentisic acid 5-O-glucoside | 1.39626 | 1.96 | 0.0010 |
| **48** | *trans*-Cinnamaldehyde | 1.38744 | -1.22 | 0.0003 |
| **42** | Cinnamyl alcohol | 1.38222 | 1.88 | 0.0008 |
| **43** | 4-Methylumbelliferyl-$\alpha$-D-glucopyranoside | 1.34671 | / | 0.1463 |
| **40** | Kaempferol 3-O-$\alpha$-L-arabinopyranosyl -7-O-$\alpha$-L-rhamnopyranoside | 1.29033 | 7.41 | 0.0055 |
| **41** | 2-Methoxybenzaldehyde | 1.23925 | Specific in CR | 0.0258 |
| **38** | Kaempferol | 1.23917 | 3.21 | 0.0116 |
| **34** | Graveobioside A | 1.22572 | 3.48 | 0.0114 |
| **5** | Catechol | 1.22299 | -0.67 | 0.0231 |
| **16** | 4-Acetyl-3-hydroxy-5-methylphenyl β-D-glucopyranoside | 1.21275 | -1.55 | 0.0067 |
| **17** | Picconioside B | 1.21051 | -2.24 | 0.0038 |
| **45** | *trans*-Cinnamic acid | 1.20494 | -1.16 | 0.0065 |
| **26** | 2-(4-hydroxyphenyl)-7-((3,4,5-trihydroxy-6-(hydroxymethy l)tetrahydro-2H-pyran-2-yl)oxy)chroman-4-one | 1.17656 | Specific in CC | 0.0191 |
| **32** | Quercetin | 1.16950 | Specific in CR | 0.0219 |
| **31** | Coumarin | 1.16629 | 1.15 | 0.0411 |
| **44** | (±)-Abscisic acid | 1.13767 | / | 0.0797 |
| **27** | Quercetin-3$\beta$-D-glucoside | 1.13751 | 2.81 | 0.0293 |
| **33** | Quercitrin | 1.13418 | Specific in CR | 0.0296 |
| **36** | Yucalexin P-17 | 1.12735 | Specific in CC | 0.0126 |
| **7** | Salicylic acid | 1.12588 | / | 0.2768 |
| **49** | 2-Methoxycinnamic acid | 1.11565 | / | 0.1049 |
| **2** | Isovanillic acid | 1.08152 | -0.38 | 0.0374 |
| **22** | (−)-Lyoniresinol | 1.07830 | / | 0.1095 |
| **19** | Taxifolin | 1.07764 | Specific in CR | 0.0295 |
| **11** | Epicatechin | 1.05657 | / | 0.1232 |
| **6** | Neochlorogenic acid | 1.04831 | / | 0.0644 |
| **14** | Catechin | 1.02725 | / | 0.1559 |
| **35** | Libertellenone B | 1.00161 | -0.98 | 0.0497 |

[a] **Fold change=$\log_2$ ($A_{CR}/A_{CC}$). $A_{CR}$ and $A_{CC}$ represent the peak areas of the compounds in CR and CC. A positive value indicated that the expression of compound in CR was higher than that in CC, while a negative value indicated that the expression of compound in CR was lower than that in CC.**

[b] **P-value < 0.05 was significant.**

**Table S3**. Results of recovery rate.

| NO. | Name | Original sample/ug | Add/ug | Found/ug | Recovery % | Mean % | RSD % |
|---|---|---|---|---|---|---|---|
| **31** | Coumarin | 0.7071 | 73.5840 | 1.4641 | 104.69% | 100.09% | 2.42% |
| | | 0.7071 | 73.5840 | 1.4173 | 98.21% | | |
| | | 0.7071 | 73.5840 | 1.4153 | 97.93% | | |
| | | 0.7071 | 73.5840 | 1.4358 | 100.77% | | |
| | | 0.7071 | 73.5840 | 1.4328 | 100.36% | | |
| **42** | Cinnamyl alcohol | 0.0366 | 0.0373 | 0.0724 | 95.96% | 98.07% | 2.77% |
| | | 0.0366 | 0.0373 | 0.0728 | 97.13% | | |
| | | 0.0366 | 0.0373 | 0.0718 | 94.50% | | |
| | | 0.0366 | 0.0373 | 0.0743 | 101.02% | | |
| | | 0.0366 | 0.0373 | 0.0734 | 98.59% | | |
| **45** | *trans*-Cinnamic acid | 0.8004 | 0.7867 | 1.5520 | 95.53% | 95.11% | 2.09% |
| | | 0.8004 | 0.7867 | 1.5443 | 94.56% | | |
| | | 0.8004 | 0.7867 | 1.5460 | 94.77% | | |
| | | 0.8004 | 0.7867 | 1.5602 | 96.58% | | |
| | | 0.8004 | 0.7867 | 1.5222 | 91.75% | | |
| **49** | 2-Methoxy cinnamic acid | 0.0080 | 0.00798 | 0.0158 | 98.00% | 98.30% | 2.23% |
| | | 0.0080 | 0.00798 | 0.0160 | 99.63% | | |
| | | 0.0080 | 0.00798 | 0.0155 | 94.18% | | |
| | | 0.0080 | 0.00798 | 0.0158 | 98.15% | | |
| | | 0.0080 | 0.00798 | 0.0160 | 100.14% | | |
| **48** | *trans*-Cinnamaldehyde | 23.2100 | 22.8000 | 45.1864 | 96.39% | 97.36% | 3.43% |
| | | 23.2100 | 22.8000 | 46.2753 | 101.16% | | |
| | | 23.2100 | 22.8000 | 45.2955 | 96.87% | | |
| | | 23.2100 | 22.8000 | 44.2427 | 92.25% | | |
| | | 23.2100 | 22.8000 | 45.2077 | 96.48% | | |

**Table S4.** Molecular docking fraction.

| NO. | Fold change[a] | Name | -CDOCKER INTERACTION ENERGY/ kJ.mol$^{-1}$ | | | |
|---|---|---|---|---|---|---|
| | | | HbA1c | PGC1$\alpha$ | SIRT3 | AMPK |
| 1 | 1.96 | Gentisic acid 5-O- glucoside | 43.8038 | 67.0292 | / | 38.5316 |
| 2 | -0.38 | Isovanillic acid | 23.2639 | 35.9152 | 26.4249 | 22.5498 |
| 3 | -0.93 | Gentisic acid | 25.395 | 36.1031 | 23.8157 | 20.9892 |
| 5 | -0.67 | Catechol | 16.6196 | 25.7563 | 18.3868 | 17.6189 |
| 8 | Specific in CC | Citrinin | 28.8092 | 52.3062 | / | 39.842 |
| 16 | -1.55 | 4-Acetyl-3-hydroxy-5-methylphenyl $\beta$-D-glucopyranoside | 43.7324 | 64.7307 | / | 45.1652 |
| 17 | -2.24 | Picconioside B | 62.5016 | / | / | 50.7858 |
| 19 | Specific in CR | Taxifolin | 39.2912 | 60.4347 | / | 49.8092 |
| 24 | 2.93 | Lyoniside | 21.3959 | 36.4075 | 27.7012 | 23.9108 |
| 26 | Specific in CC | 2-(4-Hydroxyphenyl)-7-((3,4,5-trihydroxy-6-(hydro xymethyl) tetrahydro-2H- pyran-2-yl) oxy) chroman-4-one | 53.7767 | 46.6389 | / | 44.2027 |
| 27 | 2.81 | 2-[1-(2H-1, 3-Benzod ioxol-5-yl)propan-2-yl] | 52.3853 | / | / | 56.3881 |
| 31 | 1.15 | Coumarin | 17.7063 | 27.0471 | 18.5035 | 14.3744 |
| 32 | Specific in CR | Quercetin | 35.5995 | 66.1113 | / | 53.0311 |
| 33 | Specific in CR | Quercitrin | 48.2373 | 28.2954 | / | 63.6781 |
| 34 | 3.48 | Graveobioside A | 63.679 | / | / | 62.8879 |
| 35 | -0.98 | Libertellenone B | 32.9261 | 36.9579 | / | 41.8519 |
| 36 | Specific in CC | Yucalexin P-17 | 35.2668 | 44.4972 | / | 28.6512 |
| 40 | 7.41 | Kaempferol 3-O-$\alpha$-L-arabin opyranosyl-7-O-$\alpha$-L-rhamnopyranoside | 64.5903 | / | / | 66.4067 |
| 41 | Specific in CR | 2-Methoxy benzaldehyde | 23.2034 | 28.5392 | 19.1136 | 14.6854 |
| 42 | 1.88 | Cinnamyl alcohol | 21.9571 | 26.9709 | 24.9043 | 21.6532 |
| 45 | -1.16 | *trans*-Cinnamic acid | 18.5375 | 37.4825 | 25.3821 | 20.5352 |
| 46 | 3.28 | 4-Phenyl-3-buten-2-one | 20.6405 | 27.6936 | 23.658 | 16.5148 |
| 48 | -1.22 | *trans*-Cinnamaldehyde | 18.8605 | 27.4942 | 22.3644 | 18.7263 |
| 49 | 1.22 | 2-Methoxycinnamic acid | 20.0461 | 26.8977 | 23.5866 | 15.9781 |
| 53 | Specific in CR | 4-Ethylbenzaldehyde | 17.9064 | 27.6039 | 23.2434 | 16.3396 |
| 58 | 3.26 | 4-Methoxychalcone | 30.8117 | 35.4958 | / | 27.9932 |

[a] Fold change=$\log_2$ ($A_{CR}$/$A_{CC}$). $A_{CR}$ and $A_{CC}$ represent the peak areas of the compounds in CR and CC. A positive value indicated that the expression of compound in CR was higher than that in CC, while a negative value indicated that the expression of compound in CR was lower than that in CC.

**Table S5.** Information on 8 batches of CR and CC.

| No. | Origins | Sample sources | Collection times | Voucher specimen No. |
|---|---|---|---|---|
| CR-1 | Guangxi | Kangmei pharmaceutical Bozhou Huatuo traditional Chinese medicine city | 2020.07 | 20210910-001 |
| CR-2 | Guangxi | Henan Zhang Zhongjing Pharmacy Co., Ltd Zhengzhou City Store | 2019.08 | 20210910-002 |
| CR-3 | Guangxi | Wuzhou city, guangxi | 2021.08 | 20210910-003 |
| CR-4 | Guangxi | Kangmei pharmaceutical Bozhou Huatuo Traditional Chinese medicine city | 2021.08 | 20210910-004 |
| CR-5 | Guangxi | Henan Zhang Zhongjing Pharmacy Co., Ltd Zhengzhou City Store | 2019.08 | 20210910-005 |
| CR-6 | Guangdong | Zhaoqing, Guangdong | 2021.09 | 20210910-006 |
| CR-7 | Guangdong | Zhongtang town, dongguan city, Guangdong | 2021.06 | 20210910-007 |
| CR-8 | Sichuan | Kangmei pharmaceutical Bozhou Huatuo traditional Chinese medicine city | 2021.08 | 20210910-008 |
| CC-1 | Guangxi | Kangmei pharmaceutical Bozhou Huatuo traditional Chinese medicine city | 2021.08 | 20210911-001 |
| CC-2 | Guangxi | Kangmei pharmaceutical Bozhou Huatuo traditional Chinese medicine city | 2022.08 | 20210911-002 |
| CC-3 | Guangxi | Guangxi | 2021.09 | 20210911-003 |
| CC-4 | Guangdong | Dongguan, Guangdong province | 2021.09 | 20210911-004 |
| CC-5 | Guangxi | Anhui bozhou economic development zone | 2021.09 | 20210911-005 |
| CC-6 | Guangxi | Henan Zhang Zhongjing Pharmacy Co., Ltd Zhengzhou City Store | 2019.08 | 20210911-006 |
| CC-7 | Guangxi | Kangmei pharmaceutical Bozhou Huatuo traditional Chinese medicine city | 2021.08 | 20210911-007 |
| CC-8 | Sichuan | Kangmei pharmaceutical Bozhou Huatuo traditional Chinese medicine city | 2021.08 | 20210911-008 |

**Identification of other constituents of CR and CC**

**S1. Identification of other phenylpropanoids and phenolic acids**

The diagnostic ions at *m/z* 163/165, 151/153 and 135/137 were indicators of phenylpropanoids and phenolic acids in literature [23,67]. Besides, we found that fragment ions at *m/z* 105/107 and 123/125 also could be diagnostic ions by statistical analyses [23,67].

Compounds **1**, **2**, **3**, **4**, **15** and **18** were filtered by *m/z* 151 or 153. Compound **2** showed a precursor ion at *m/z* 167.0342 $[M{-}H]^-$, which suggested a molecular formula of $C_8H_8O_4$, and showed the fragment ions at *m/z* 151.0226 $[M - O]^-$ and *m/z* 123.0449 $[M - COOH - H]^-$ that indicated the hydroxyl and carboxyl groups, thus compound **2** putatively was identified as isovanillic acid by the published literature [29] and database. Compound **4** possessed a molecular formula of $C_8H_8O_4$ by a precursor ion at *m/z* 199.0588 $[M + H]^+$, and exhibited the fragment ions at *m/z* 181.0495$[M - OH + H]^+$, *m/z* 163.1478 $[M - 2H_2O + H]^+$ and *m/z* 153.0764 $[M - OH - OCH_3 + H]^+$, thus compound **4** was tentatively identified as syringic acid by the published literature [35]. Compounds **15** and **18** had a same molecular formula of $C_8H_8O_3$ by their same precursor ions at *m/z* 153.0545 $[M + H]^+$. However, compound **15** directly lost one carbonyl group to form an ion peak of *m/z* 125.0595 $[M - CO + H]^+$ and compound **18** lost one carboxyl and methylene group and showed *m/z* 95.0491 $[M - CO_2 - CH_2 + H]^+$, thus compounds **15** and **18** were respectively presumed to be vanillin [39] and 2-anisic acid [68]. Compounds **1** and **3** with the molecular formulas of $C_{13}H_{16}O_9$, and $C_7H_6O_4$ by the precursor ions at *m/z* 315.0709 $[M + H]^+$ and 153.0187 $[M + H]^+$, had the common fragment ions at *m/z* 153.0185 $[M - C_6H_{10}O_5 + H]^+$ or 153.0187 $[M + H]^+$, and *m/z* 109.0293 $[M - C_6H_{10}O_5 - COOH + H]^+$ or *m/z* 109.0288 $[M - COOH + H]^+$, thus compounds **1** and **3** were tentatively identified as gentisic acid 5-O-glucoside and gentisic acid [35].

Compounds **9**, **41**, **45**, **46**, **48**, **49**, **52**, **53** and **55** were filtered by *m/z* 105 or 107. Compound **46** possessed a molecular formula of $C_{10}H_{10}O$ by the ion peak at *m/z* 147.0803 $[M + H]^+$, and also showed the fragment ions at *m/z* 132.0567 $[M - CH_3 + H]^+$, *m/z* 107.0489 $[M - CH - CO + H]^+$ and *m/z* 91.0541 $[M - C_3H_4O + H]^+$, thus compound **46** was tentatively identified as 4-phenyl-3-buten-2-one [49]. Compound **49** produced the ion peak at *m/z* 161.0596 $[M - H_2O + H]^+$, indicating a molecular formula of $C_{10}H_{10}O_3$, and also showed the fragment ions at *m/z* 133.1011 $[M - H_2O - CO + H]^+$, *m/z* 119.0855 $[M - CHO - OCH_2 + H]^+$ and *m/z* 105.0698 $[M - COOH - CH + H]^+$, thus compound **49** was putatively

identified as 2-methoxycinnamic acid [48]. Compound **52** with a molecular formula of $C_{13}H_{18}O_4$ by *m/z* 239.1275 $[M + H]^+$, showed the signals at *m/z* 205.1192 $[M - 2OH + H]^+$, *m/z* 179.0705 $[M - C_2H_5 - OCH_3 + H]^+$, *m/z* 151.0753 $[M - C_2H_5 - C_3H_7O + H]^+$, *m/z* 107.0481 $[M - 2OH - 2OCH_3 - C_2H_4 - C_3H_3 + H]^+$ and *m/z* 95.0861 $[M - OH - 2OCH_3 - C_2H_5 - C_4H_3O + H]^+$, thus compound **52** was putatively identified as deoxyphomalone [50]. Compound **55** with a molecular formula of $C_{10}H_{10}O_2$ by *m/z* 163.0754 $[M + H]^+$, and exhibited the ions at *m/z* 135.0803 $[M - CO + H]^+$, *m/z* 110.0203 $[M - C_3H_3O + H]^+$, *m/z* 105.0699 $[M - CO - OCH_3 + H]^+$ and *m/z* 79.0542$[M - C_3H_3O - OCH_3 + H]^+$, thus compound **55** was putatively identified as 4-methoxy cinnamaldehyde [46]. The cleavage of compounds **45** and **48** were shown in the manuscript.

Compounds **9** and **41** with a common molecular formula of $C_8H_8O_2$ showed the similar fragment ions at *m/z* 107, 93, 79. For example, compound **9** with $[M + H]^+$ ions of *m/z* 137.0596 produced an $[M - OCH_2 + H]^+$ ion at *m/z* 107.0490, an $[M - CO - CH_3 + H]^+$ ion at *m/z* 93.0590, and an $[M - CO - OCH_3 + H]^+$ ion at *m/z* 79.0543. Thus compounds **9** and **41** were tentatively identified as 4-methoxybenzaldehyde and 2-methoxybenzaldehyde [28]. Compound **53** produced *m/z* 135.0803 $[M+H]^+$, with a formula of $C_9H_{10}O$, and also showed *m/z* 135.0803 $[M + H]^+$, *m/z* 107.0490 $[M - CO + H]^+$, *m/z* 105.0697 $[M - C_2H_6 + H]^+$ and *m/z* 79.0542 $[M - C_2H_6 - CO + H]^+$, thus compound **53** was presumed to be 4-ethylbenzaldehyde [52].

Compound **6** showed the precursor ion at *m/z* 353.0886 $[M - H]^-$, which indicated a formula of $C_{16}H_{18}O_9$, was tentatively identified as neochlorogenic acid [35], it produced similar fragment ions at *m/z* 191.0550 $[M - C_9H_6O_3 - H]^-$, *m/z* 179.0343 $[M - C_7H_{10}O_5 - H]^-$, *m/z* 161.0237 $[M - C_7H_{10}O_5 - H_2O - H]^-$ and *m/z* 135.0445 $[M - C_7H_{10}O_5 - CO_2 - H]^-$. Compound **7** had the precursor ion at *m/z* 137.0238 $[M - H]^-$, with a formula of $C_7H_6O_3$, and also showed *m/z* 119.0132 $[M - H_2O - H]^-$, *m/z* 108.8992 $[M - CO - H]^-$ and *m/z* 93.0343 $[M - COO - H]^-$, thus compound **7** presumed to be vanillin salicylic acid [24].

Compound **16** had a molecule formula of $C_{15}H_{20}O_8$ by *m/z* 327.1073 $[M-H]^-$, showed the fragment ions at *m/z* 165.0548 $[M - C_6H_{10}O_5 - H]^-$, *m/z* 147.0446 $[M - C_6H_{10}O_5 - H_2O - H]^-$ and *m/z* 121.0653 $[M - C_6H_{10}O_5 - OH - CH_3 - CH_2 - OH]^-$, therefore compound **16** were tentatively identified as 4-acetyl-3-hydroxy- 5-methylphenyl-*β*-D-glucopyranoside [27]. Compound **21** produced a molecule formula of $C_8H_{10}O$ by *m/z* 121.0654 $[M - H]^-$ and *m/z* 106.0419 $[M - CH_3 - H]^-$, which tentatively identified compound **21** as 4-ethylphenol [44]. Compound **25** showed the ions at *m/z*

165.0548 $[M - H]^-$, which suggested a molecule formula of $C_{15}H_{20}O_8$, and showed *m/z* 136.9315 $[M - OCH_3 - H]^-$ and *m/z* 96.9597 $[M - C_2H_3O_2 - H]^-$, hence compound **25** was tentatively identified as 3-methoxyphenylacetic acid by the published literature and database [69]. Compound **42** had a formula of $C_9H_{10}O$, and exhibited the ions at *m/z* 117.0698 $[M - H_2O + H]^+$, and *m/z* 91.0540 $[M - C_2H_4O + H]^+$, thus compound **42** was identified as cinnamyl alcohol [46].

**S2. Identification of other flavonoids**

Two epicatechins **11** and **14** were filtered by *m/z* 291 or 289 [23,67]. Compounds **11** and **14** showed a same molecular formulas $C_{15}H_{14}O_6$ through their precursor ions at *m/z* 291.0860 $[M + H]^+$ and *m/z* 289.0703 $[M - H]^-$, respectively. And their fragmentation behaviours and fragment ions were basically identical, thus compound **14** should be an isomer of **11** that was identified by the retention time of standard. Therefore, compound **14** were presumed to be catechin [70,71].

Three quercetins **27**, **32** and **33** were filtered by *m/z* 303 or 301 [23,67]. Compounds **27**, **32** and **33** had the molecular formulas of $C_{21}H_{20}O_{12}$, $C_{15}H_{10}O_7$ and $C_{21}H_{20}O_{11}$, but showed the similar fragment ions at *m/z* 257, 229. For example, compound **32** with $[M + H]^+$ ions of *m/z* 303.0496 produced an $[M - CO - H_2O + H]^+$ ion at *m/z* 257.0446 and an $[M - 2CO - H_2O + H]^+$ ion at *m/z* 229.0491, thus compound **32** was identified as quercetin by a reference standard. Compound **33** was presumed to be quercitrin by a neutral loss of rhamnose moiety at *m/z* 303.0496 $[M + H - C_6H_9O_4]^+$ in the $MS^2$ spectrum, and compound **27** with a neutral loss of a glucose moiety *m/z* 301.0338 $[M - H - C_6H_{10}O_5]^-$, tentatively identified compound **27** as quercetin-3*β*-D-glucoside [34].

Two kaempferols **38** and **40** were filtered by *m/z* 287 or 285 [23,67]. The molecular formula of compound **38** was $C_{15}H_{10}O_6$ by its precursor ion at *m/z* 287.0550 $[M + H]^+$, and it has been identified as kaempferol by combining the mass fragments of literatures and standard. Compound **40** was presumed to be kaempferol 3-O-*α*-L-arabinopyranosyl-7-O-*α*-L-rhamnopyranoside[23,67, 70, 71]. It presented a formula of $C_{26}H_{28}O_{14}$ and showed a neutral loss of a glucose moiety and an arabinopyranosyl moiety *m/z* 284.0316 $[M - C_6H_8O_4 - C_5H_{10}O_4 - H]^-$.

Two dihydroflavones **19** and **26** were identified. Compound **19** showed a precursor ion *m/z* 305.0662 $[M + H]^+$ and its molecular formula was $C_{15}H_{12}O_7$, and also showed *m/z* 287.0573 $[M - H_2O + H]^+$, *m/z* 257.0591 $[M - CO - H_2O + H]^+$ and *m/z* 153.0188 $[M - CO - C_7H_8O_2 + H]^+$, thus compound **19** was putatively identified as taxifolin [12]. Compound **26** produced a precursor ion *m/z* 417.1177 [M +

H]$^+$ that suggested a molecular formula of $C_{21}H_{22}O_9$, and also showed a neutral loss of a glucose moiety *m/z* 301.0338 [M − $C_6H_{10}O_5$ − H]$^-$ and 153.0187 [M − $C_6H_{10}O_5$ – CO − $C_7H_6O_2$ − H]$^-$, thus compound **26** was putatively identified as 2-(4-hydroxyphenyl)-7-((3,4,5-trihydroxy-6-(hydroxymethyl) tetrahydro-2H-pyran-2 -yl) oxy) chroman-4-one [67, 12].

**S3. Identification of other coumarin**

Compound **43** showed the similar fragmentation behaviours and fragment ions as **31**, but compound **43** had a molecular formula of $C_{16}H_{18}O_8$ by a precursor ion at *m/z* 339.1075 [M + H]$^+$, and showed a neutral loss of a glucose moiety and a $CH_3$ that formed *m/z* 177.0546 [M + H − $C_6H_{10}O_5$]$^+$ and *m/z* 145.0284 [M − $C_6H_{10}O_5$ − $CH_3$ – OH + H]$^+$, thus compound **43** was tentatively identified as 4-methylumbelliferyl-$\alpha$-D- glucopyranoside by database.

**S4. Identification of other Lignans**

Three lignans (**20**, **22** and **23**) were filtered by *m/z* 419 or 420 [21]. Compounds **20**, **22** and **23** showed precursor ions *m/z* 581.2209 [M − H]$^-$, *m/z* 419.1694 [M − H]$^-$ and 551.2105 [M − H]$^-$, which suggested molecular formulas of $C_{28}H_{38}O_{13}$, $C_{22}H_{28}O_8$ and $C_{27}H_{36}O_{12}$, and showed the filtering ions at *m/z* 419.1691 [M − $C_6H_{10}O_5$ − H]$^-$, *m/z* 419.1694 [M − H]$^-$ and *m/z* 419.1650 [M − $C_5H_{10}O_4$ − H]$^-$ and showed the common ions at *m/z* 373.1272 [M − $C_6H_{10}O_5$ − 3$CH_3$ − H]$^-$ and 359.1110 [M − $C_6H_{10}O_5$ − 4$CH_3$ − H]$^-$, or *m/z* 373.1277 [M − 3$CH_3$ − H]$^-$ and *m/z* 359.1119 [M − 4$CH_3$ − H]$^-$, or *m/z* 373.1272 [M − $C_6H_{10}O_5$ − 3$CH_3$ − H]$^-$ and *m/z* 359.1110 [M − $C_6H_{10}O_5$ − 4$CH_3$ − H]$^-$, hence compounds **20**, **22** and **23** were tentatively identified as lyoniresinol-3a-O-$\beta$-D-glucopyranosid, (-)-lyoniresinol and lyoniside [33,42].

Compound **28** had a molecular formula of $C_{20}H_{22}O_4$ by the ion peak at *m/z* 327.1593 [M + H]$^+$, and showed the fragment ions at *m/z* 295.1328 [M − $OCH_2$ + H]$^+$ and *m/z* 163.0753 [M − $C_{10}H_{12}O_2$ + H]$^+$, thus compound **28** was tentatively identified as 2-[1-(2H-1, 3-benzod ioxol-5-yl) propan-2-yl]-6-methoxy-4-(prop-2-en-1-yl) phenol [33,42].

**S5. Identification of terpenoids**

A total of six terpenoids were discovered, including compounds **35**, **36**, **44**, **57**.

Compound **35** was tentatively identified as libertellenone B. It presented a formula of $C_{20}H_{26}O_4$ and produced several main fragment ions at *m/z* 331.1902 [M + H]$^+$, *m/z* 313.1796 [M − $H_2O$ + H]$^+$, *m/z* 165.0911 [M − $C_{10}H_{14}O_2$ + H]$^+$ and *m/z* 125.0565 [M − $C_{12}H_{14}O_3$ + H]$^+$. Compound **36** possessed a molecular formula of $C_{17}H_{20}O_3$ by the ion peak at *m/z* 273.1488 [M + H]$^+$, *m/z* 255.1391 [M − $H_2O$ + H]$^+$, and showed *m/z* 227.1441 [M − $H_2O$ – CO + H]$^+$, *m/z* 149.0964 [M − $CH_3$ − $C_6H_6O_2$ + H]$^+$ and *m/z* 82.8045 [M − $C_{11}H_{10}O_3$ + H]$^+$, thus compound **36** was tentatively identified as 3(2H)-phenanthrenone, 1, 9, 10, 10a-tetrahydro-2, 6-dihydroxy-1, 1, 7-trimethyl by the mass information of database. Compound **44** with a molecular formula of $C_{15}H_{20}O_4$ by *m/z* 265.1481 [M + H]$^+$, produced the fragment ions at *m/z* 247.1332 [M − $H_2O$ + H]$^+$, *m/z* 229.1216 [M − $2H_2O$ + H]$^+$ and *m/z* 187.1108 [M – O − $CH_2$ – COO + H]$^+$, thus compound **44** was tentatively identified as (±)-abscisic acid by the mass information of database. Compound **57** had a molecular formula of $C_{15}H_{20}O_4$ by the ion peak at *m/z* 221.1900 [M + H]$^+$, and showed the fragment ions at *m/z* 175.1483 [M – O − $2CH_2$ – C + H] $^+$, *m/z* 161.1323 [M − $2CH_3$ – C – CH – O + H]$^+$ and *m/z* 147.1167 [M − $2CH_3$ – C – CH − $CH_2$ – O + H]$^+$. Therefore, compound **57** was tentatively identified as (-)-caryophyllene oxide by the mass information of database.

**S6. Identification of organic acids**

A total of 11 organic acids were discovered, including compounds **8**, **13**, **17**, **24**, **37**, **39**, **47**, **50**, **51**, **56**. Compound **8** had a molecular formula of $C_{13}H_{14}O_5$ by the ion peak at *m/z* 251.0915 [M + H]$^+$, and showed *m/z* 233.0804 [M − $H_2O$ + H]$^+$, *m/z* 221.0807 [M − $2CH_3$ + H]$^+$ and *m/z* 205.0857 [M − $H_2O$ – CO + H]$^+$, thus compound **8** was putatively identified as citrinin by the mass information of database. Compound **17** showed a molecular formula of $C_{26}H_{40}O_{12}$ by the ion peak at *m/z* 543.2421 [M − H]$^-$, and showed *m/z* 525.2305 [M − $H_2O$ − H]$^-$ and 363.1800 [M − $H_2O$ − $C_6H_{10}O_5$ − H]$^-$, thus compound **17** should be picconioside B by the mass information of database. Compound **24** had the ion peak at *m/z* 177.0545 [M + H]$^+$, which indicated a molecular formula of $C_{10}H_8O_3$ and showed *m/z* 149.0596 [M – CO + H]$^+$, *m/z* 133.0646 [M – COO + H]$^+$, *m/z* 105.0693 [M – CO – COO + H]$^+$, *m/z* 93.0098 [M – CO – COO – C + H]$^+$ and *m/z* 81.0700 [M – CO – COO − 2C + H]$^+$, thus compound **24** was putatively identified as 3-oxoindane-1-carboxylic acid by the mass information of database. Compound **37** showed the precursor ion at *m/z* 187.0966 [M − H]$^-$, which indicated a formula of $C_9H_{16}O_4$ , was tentatively identified as 3-oxoindane-1-carboxylic acid [47], it produced similar fragment ions at *m/z* 125.0966 [M – COOH – OH − H]$^-$ and *m/z* 97.0654 [M − 2COOH − H]$^-$. Compound **39** possessed a

molecular formula of $C_9H_{14}O_4$, and showed the ion peaks at *m/z* 185.0819 [M − H]$^-$, *m/z* 141.0916 [M – COO − H]$^-$ and *m/z* 104.0775 [M − $C_6H_9$ − H]$^-$, thus compound **39** was tentatively identified as 1-(carboxymethyl) cyclohexanecarboxylic acid by the mass information of database. Compound **47** showed the precursor ion at *m/z* 201.1120 [M − H]$^-$, with a molecular formula of $C_{10}H_{18}O_4$ by , and showed *m/z* 183.1020 [M – OH − H]$^-$ and *m/z* 156.8982 [M – COO − H]$^-$, hence compound **47** was putatively identified as 3-tert-butyladipic acid by the mass information of database. Compound **50** was tentatively identified as 9*S*, 13*R*-12-oxophytodienoic acid [72]. It presented a formula of $C_{18}H_{28}O_3$ and produced several main fragment ions at *m/z* 293.2104 [M + H]$^+$, *m/z* 275.2003 [M − $H_2O$ + H]$^+$, *m/z* 239.1799 [M − $C_4H_6$ + H]$^+$, *m/z* 147.1163 [M − $C_7H_{14}$ + H]$^+$ and *m/z* 81.0698 [M − $C_{12}H_{20}O_3$ + H]$^+$. Compound **51** had the ion peak at *m/z* 327.2165 [M − H]$^-$, which suggested a molecular formula of $C_{18}H_{32}O_5$, and showed *m/z* 242.9845 [M − $C_5H_4$ – OH − H]$^-$, *m/z* 171.1019 [M − $C_9H_{16}O_2$ − H]$^-$ and *m/z* 85.0291 [M − $C_{13}H_{22}O_4$ − H]$^-$, thus compound **51** was tentatively identified as corchorifatty acid F [51]. The mass fragmentation of compound **56** was basically accordant with that of **51**, except that all the ion peaks of **56** were 2 *m/z* value larger than those of **51**, which indicated a less of one double bond. Thus, compound **56** was tentatively identified as 3-tert-butyladipic acid [51].

**S7. Identification of others**

Compound **12** was identified as 2, 4, 6-trihydroxy-2-(4-hydroxybenzyl) -1-benzofuran-3(2H)-one with *m/z* 287.0551 [M − H]$^-$ ($C_{15}H_{12}O_6$), and fragmentions at *m/z* 161.0233 [M − $C_6H_6O_3$ − H]$^-$ and *m/z* 125.0239 [M − $C_8H_2O_4$ − H]$^-$. Compound **30** was tentatively identified as 6-hydroxy-8-methoxy-3-methyl-3, 4-dihydro -1H-isochromen-1-one. It presented a formula of $C_{11}H_{12}O_4$ and produced several main fragment ions at *m/z* 209.0802 [M + H]$^+$, *m/z* 149.0596 [M – COOCH + H]$^+$ and *m/z* 121.0647 [M − 2OH − $OCH_3$ – CO + H]$^+$. Compound **34** had a molecular formula of $C_{26}H_{28}O_{15}$ by the ion peak at *m/z* 579.1325 [M − H]$^-$, and exhibited *m/z* 417.1531 [M − $C_6H_{10}O_5$ − H]$^-$, *m/z* 300.0259 [M − $C_6H_8O_4$ − $C_5H_{10}O_4$ − H]$^-$, *m/z* 271.0235 [M − $C_{11}H_{16}O_{10}$ − H]$^-$ and *m/z* 178.9979 [M − $C_{17}H_{20}O_{11}$ − H]$^-$, thus compound **34** was tentatively identified as graveobioside A [42]. Compound **54** had a molecular formula of $C_{10}H_8O$ by the ion peak at *m/z* 145.0647 [M + H]$^+$, showed *m/z* 116.0575 [M – C – OH + H]$^+$, *m/z* 102.0468 [M − $C_2H_2$ – OH + H]$^+$ and *m/z* 91.0539 [M − $C_3H_2$ – OH + H]$^+$, thus compound **54** was tentatively identified as 1-naphthol by the published literature [53]. Compound **58** was tentatively identified as 4-methoxychalcone [73]. It presented a formula of

$C_{16}H_{14}O_2$ and produced several main fragment ions at *m/z* 239.1073 $[M + H]^+$, *m/z* 161.0595 $[M - C_6H_6 + H]^+$, *m/z* 133.0647 $[M - C_7H_6O + H]^+$ and *m/z* 105.0333 $[M - C_6H_6 - C_2H - OCH_3 + H]^+$.
